# Extreme Urban Heat and Emergency Department Visits in Older Adults

**DOI:** 10.1001/jamanetworkopen.2026.2645

**Published:** 2026-03-20

**Authors:** Evan Siau, Genevieve S. Silva, Jeremy Lu, Cassandra Thiel, Simon Jones, Leora I. Horwitz, Katie E. Lichter, Alexander Azan

**Affiliations:** 1 Department of Medicine, New York University Grossman School of Medicine, New York; 2 Department of Population Health, New York University Grossman School of Medicine, New York; 3 Transitional Year Residency Program, Penn Medicine Doylestown, Doylestown, Pennsylvania; 4 Department of Radiation Oncology, University of California, San Francisco; 5 Department of Radiation Oncology and Applied Sciences, Dartmouth, Lebanon, New Hampshire; 6 The Dartmouth Institute, Dartmouth, Lebanon, New Hampshire

## Abstract

**Importance:**

Health care systems can help protect patients from the increasing threat of extreme heat-driven morbidity and mortality. Electronic health records (EHRs) provide insight into trends and local variation in thresholds above which extreme heat is associated with emergency department (ED) use among at-risk patient populations.

**Objective:**

To examine associations between extreme heat exposure and all-cause ED visits among patients aged 65 years and older.

**Design, Setting, and Participants:**

This matched case-control study of patients seeking emergency care at an urban health care system during the summer (May 1 to September 30) from 2022 to 2024. Two New York City (NYC) EDs were included: (1) ED-1, predominantly serving Medicaid-enrolled patients from minoritized racial and ethnic groups, and (2) ED-2, predominantly serving White, privately insured patients. Included patients were aged 65 years or older and presented to ED-1 and ED-2 during the study period. Data were analyzed from April to August 2025.

**Exposures:**

Daily maximum heat index (HI_max_) values during the summer were calculated from the National Centers for Environmental Information monitor-derived recordings.

**Main Outcomes and Measures:**

Daily all-cause ED use counts were derived from EHRs, and extreme heat exposure–outcome curves were calculated. Daily HI_max_ anomalies were calculated based on a 30-year baseline average. The cumulative odds ratio (OR) and 95% CIs were calculated.

**Results:**

This study included 55 200 ED encounters and represented 15 092 unique patients at ED-1 and 19 559 at ED-2 with a mean (SD) age of 74.9 (8.92) years at ED-1 and 74.9 (8.72) years at ED-2. Compared with ED-2, more ED-1 patients were female (8589 [56.9%] vs 10 767 [55.0%]), Hispanic (3544 [23.5%] vs 2576 [13.2%]), and Medicaid-enrolled (1321 [8.8%] vs 824 [4.2%]). At ED-1, daily HI_max_ associations increased after 66 °F (OR, 1.10 [95% CI, 1.01-1.21]), peaking at 101 °F (OR, 1.24 [95% CI, 1.11-1.39]), and were higher on days with HI_max_ anomalies between 15 °F (OR, 1.07 [95% CI, 1.01-1.13]) and 18 °F (OR, 1.10 [95% CI, 1.01-1.20]) warmer than average. At ED-2, daily HI_max_ ED use associations were not significant and were significantly negative for days with HI_max_ anomalies above 16 °F, nadiring at 21 °F (0.84, 95% CI [0.73, 0.95]) warmer than average.

**Conclusions and Relevance:**

In this case-control study of the association between heat exposure and ED use in adults aged 65 years and older, positive associations were only observed at ED-1, which served a predominantly lower-income population from minoritized racial and ethnic groups. These association thresholds were not fully captured by NYC heat advisories, which were triggered by 2 days above HI_max_ 95 °F or any time above 100 °F, highlighting an opportunity for future research to develop targeted, risk-informed health care system-based heat warning strategies.

## Key Points

**Question:**

Is extreme heat associated with emergency department (ED) use among urban-dwelling older adults?

**Findings:**

In this matched case-control study of 34 651 patients aged 65 years and older in an urban health care system, associations between summer heat and EHR-derived all-cause ED use varied by site. At ED-1 (an ED with more Medicaid-enrolled patients from minoritized racial and ethnic groups), use increased with higher daily maximum and anomalous heat indices, which was not observed at ED-2 (an ED with more White, privately insured patients).

**Meaning:**

These findings suggest that significant heat-health risks were observed in an ED serving socioeconomically vulnerable populations, suggesting tailored heat warning strategies should be studied further.

## Teaser

This case-control study examines associations of heat exposure and all-cause emergency department (ED) visits among patients aged 65 years and older in New York City.

## Introduction

In New York City (NYC), prior citywide epidemiologic studies have informed reductions in municipal heat advisory thresholds, contributing to measurable declines in heat-related hospitalizations.^1^ Electronic health record (EHR) data offer a valuable opportunity for health care systems to identify heat exposure thresholds associated with acute health care use, which may differ from population-level trends that inform municipal warning systems.^2^ For example, EHR–informed heat exposure thresholds may vary across a health care system’s practice settings based on differences in social and structural risk factors represented within their catchment areas.^3,4,5^ In this way, health care system-derived thresholds can be leveraged to inform tailored, heat-adaptive strategies for uniquely vulnerable patients.^6^

We examined the association between extreme heat and emergency department (ED) visits among older adults, who are especially vulnerable to heat, at 2 EDs within a large NYC health care system.^7,8^ We descriptively compared daily maximum heat index (HI_max_) values associated with increased ED use to the current NYC heat advisory threshold to illustrate the potential utility of future research and development of a health care system-based heat warning system.^9^ We also examined associations using daily HI_max_ anomalies as this metric captures exposure to heat that populations are less acclimated to, potentially posing greater health risks.^10^

## Methods

This study received approval from the New York University Grossman School of Medicine institutional review board. This case-control study was conducted and reported in accordance with the Strengthening the Reporting of Observational Studies in Epidemiology (STROBE) reporting guideline.^11^ Informed consent was not required due to the use of deidentified data.

### Study Population

We included patients aged 65 years and older who presented to 2 urban EDs within the same health care system, ED-1 and ED-2, during the summer (ie, May 1 to September 30) from 2022 to 2024. ED-1 is a safety-net, community-based academic hospital that serves a socioeconomically, racially, and ethnically diverse patient population. ED-2 is based within a larger academic medical center that primarily serves a higher-income patient population. The shortest road-network (driving) distance between ED-1 and ED-2 is approximately 10 miles. Patient sex, age, self-reported race and ethnicity, and insurance type was classified and reported as documented in EHRs.

### Temperature and Health Data

Monitor-derived hourly ambient temperature and relative humidity records were obtained from the National Centers for Environmental Information. We selected temperature data from the LaGuardia Airport monitoring site, which is highly correlated with the other major NYC National Weather Service sites (JFK International Airport, Central Park, Newark International Airport).^12^ Daily HI_max_ values were calculated using the National Weather Service equation.^13^ We operationalized HI_max_ values over other extreme heat metrics to allow for direct comparison with NYC Heat Advisory thresholds (2 consecutive days of HI_max_ 95 °F or higher, or any length of time with HI 100 °F or higher).^9^ Daily HI_max_ anomalies were calculated by comparing daily values with a 30-year baseline (1992 to 2021).

We tabulated EHR-derived, daily all-cause ED use counts (*International Statistical Classification of Diseases and Related Health Problems, Tenth Revision* codes). Repeat ED encounters were included as individual visits.

### Statistical Analysis

We leveraged a time-stratified, case-crossover design, in which each patient served as their own control, to examine the association between daily HI_max_ or HI_max_ anomalies and all-cause ED visits. Exposure on case dates was compared with exposure on control dates for each patient, matched by day of week, month, and year to account for time-invariant covariates and long-term trends (eg, seasonality, day-of-week), generating 3 or 4 control dates per case date.^14,15^ We fitted conditional logistic regression models with nonlinear functions to account for potential nonlinear effects on our outcome with time-series exposure data.^16^ Consistent with prior environmental health research,^17^ HI_max_ was modeled as a natural cubic spline with 4 degrees of freedom, with internal knots equally spaced across the observed exposure distribution. To assess the robustness of the shape of estimated exposure-response curves, sensitivity analyses were conducted by varying spline flexibility (2 to 6 degrees of freedom), refitting models with alternative internal knot placements (tenth, fiftieth, and ninetieth percentiles and twenty-fifth, fiftieth, and seventy-fifth percentiles) while holding degrees of freedom constant, and estimating models including a quadratic term for HI_max_ to represent a parsimonious nonlinear specification. We did not model distributed lag functions because no significant lagged impacts were observed in performed autocorrelative function analyses (eFigure 1 in Supplement 1). Results were calculated as odds ratios (ORs) representing the relative effect size of an elevated HI_max_, compared with a reference value, on all-cause ED visits. For HI_max_ models, the reference (56.1 °F) was the lowest HI_max_ of minimum effect, including all case days.^18^ For anomaly models, the reference HI_max_ value was 0, representing the absence of HI_max_ departures from a 30-year baseline. Models were stratified by ED site. We used descriptive statistics to quantify the number of days meeting absolute or anomaly HI_max_ thresholds that were positively associated with ED use yet did not trigger a NYC heat advisory. We did not account for acute changes in ambient air pollution in our models, as low correlation coefficients (ie, less than 0.08) were observed between daily air quality index and all-cause ED visits (eFigure 2 in Supplement 1).

We performed additional sensitivity analyses that (1) excluded patients seen at both ED sites during the study period, (2) substituted daily maximum ambient temperature (T_max_) as the exposure, and (3) stratified models by patient sex, age, self-reported race and ethnicity, and insurance type. Quantitative comparisons across strata were not reported due to limited sample sizes within various strata, reflecting the study’s single health care system setting. Analyses used R version 4.5.0 (R Project for Statistical Computing).

To provide an illustrative estimate of the potential impact of a health care system-based heat warning system, we estimated model-based heat-attributable ED visits using mean attributable fractions derived from conditional logistic regression models.^19,20^ Event days were included if the date did not trigger a municipal heat advisory and was identified as having elevated risk of heat-associated ED use based on model-derived exposure-response estimates for HI_max_ and HI_max_ anomaly across study sites. ORs corresponding to each day’s observed exposure relative to the reference temperature were treated as approximations of relative risks. For each included day, an attributable fraction was calculated as (OR − 1)/OR × observed mean number of ED visits that day. Attributable visits were summed across days. We then applied an external effectiveness estimate of 32%, based on prior evaluation of NYC heat advisory threshold changes, to estimate the number of potentially preventable ED visits.^1^ These estimates are intended as rough, illustrative approximations rather than causal estimates.

Data were analyzed from April to August 2025. Statistical significance was set at α .05, and all tests were 2-sided.

## Results

Our study population included 55 200 ED visits, representing 15 092 unique patients at ED-1 (27.3%), and 19 559 at ED-2 (35.4%). At ED-1, patients had a mean (SD) age of 74.9 (8.92) years and 8589 were female (56.9%), 2191 were Asian (14.5%), 1206 were Black (8.0%), 3544 were Hispanic or Latino (23.5%), 7211 were White (47.8%), and 27 identifed as Other (ie, American Indian or Alaska Native and Native Hawaiian or Other Pacific Islander) (0.2%). At ED-2, patients had a mean (SD) age of 74.9 (8.72) years and 10 767 were female (55.0%), 1472 were Asian (7.5%), 2013 were Black (10.3%), 2576 were Hispanic or Latino (13.2%), 12 098 were White (61.9%), and 55 identified as Other (0.3%). Compared with ED-2, more ED-1 patients had Medicaid (1321 [8.8%] vs 824 [4.2%]) (Table). A total of 564 patients presented to both EDs and were counted as unique visits at each site in primary analyses.

**Table.** Demographics of Patients Aged 65 Years or Older Seen at ED-1 and ED-2 From May to September 2022 to 2024

Characteristic	Patients, No. (%)	
	ED-1 (n = 15 092)	ED-2 (n = 19 559)
Sex		
Female	8589 (56.9)	10 767 (55.0)
Male	6502 (43.1)	8790 (44.9)
Unknown	1 (0)	2 (0)
Age, mean (SD)	74.9 (8.92)	74.9 (8.72)
Race and ethnicity		
Hispanic or Latino	3544 (23.5)	2576 (13.2)
Non-Hispanic		
American Indian or Alaska Native	17 (0.1)	34 (0.2)
Asian	2191 (14.5)	1472 (7.5)
Black or African American	1206 (8.0)	2013 (10.3)
Native Hawaiian or other Pacific Islander	10 (0.1)	21 (0.1)
White	7211 (47.8)	12 098 (61.9)
Not specified	913 (6.0)	1345 (6.9)
Insurance		
Medicaid	1321 (8.8)	824 (4.2)
Medicare	11 928 (79.0)	15 974 (81.7)
Missing	396 (2.6)	272 (1.4)
Private	1447 (9.6)	2489 (12.7)

Abbreviation: ED-1, emergency department predominantly serving Medicaid-enrolled patients from minoritized racial and ethnic groups; ED-2, emergency department level 2 predominantly serving White, privately insured patients.

**Figure 1.**
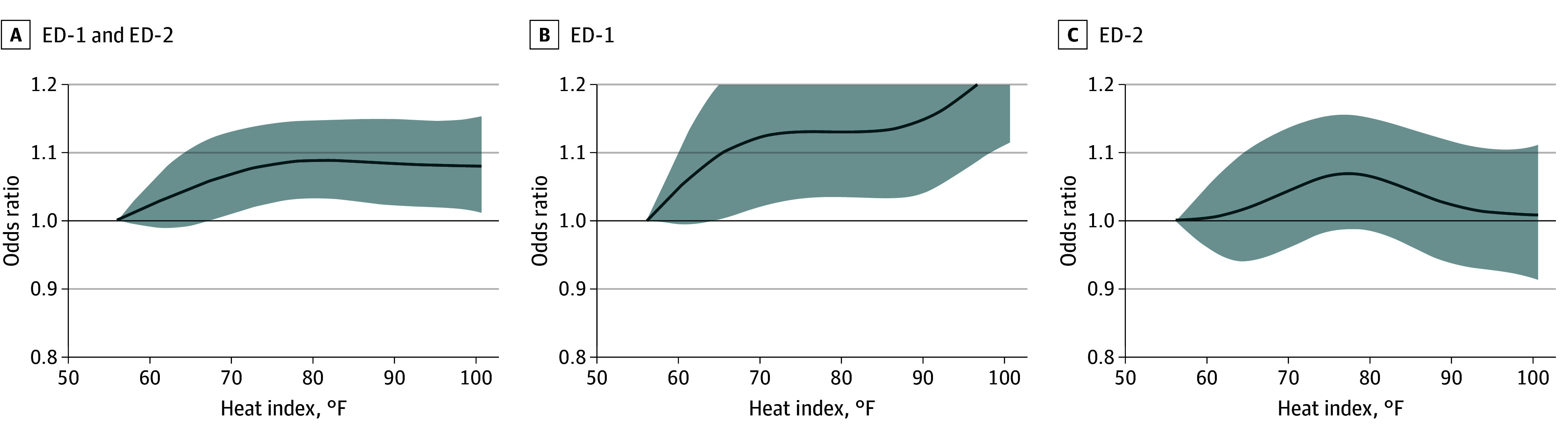
Graph of Cumulative Odds Ratios (ORs) and 95% CIs of All-Cause Emergency Department (ED) Visits From May to September 2022 to 2024, Among Adults 65 Years or Older as a Function of Daily Maximum Heat Index (HI_max_) Compared With the Reference Lowest HI_max_ of Minimum Effect (56.1 °F) OR is displayed on the y-axis, and HI_max_ is displayed on the x-axis. Panel A represents data from both ED sites combined, Panel B represents ED-1 only, and Panel C represents ED-2 only. Dark gray curves indicate cumulative odds, and gray shading indicates 95% CIs. ED-1 indicates EDs predominantly serving Medicaid-enrolled patients from minoritized racial and ethnic groups, and ED-2 indicates EDs predominantly serving White, privately insured patients.

At ED-1, utilization odds significantly increased starting at 66 °F (OR, 1.10 [95% CI, 1.01-1.21]), with amplified risks observed between 90 °F (OR, 1.15 [95% CI, 1.04-1.27]) and 101 °F (OR, 1.24 [95% CI, 1.11-1.39]) (Figure 1 and eTable 1 in Supplement 1). At ED-2, no significant associations were observed.

**Figure 2.**
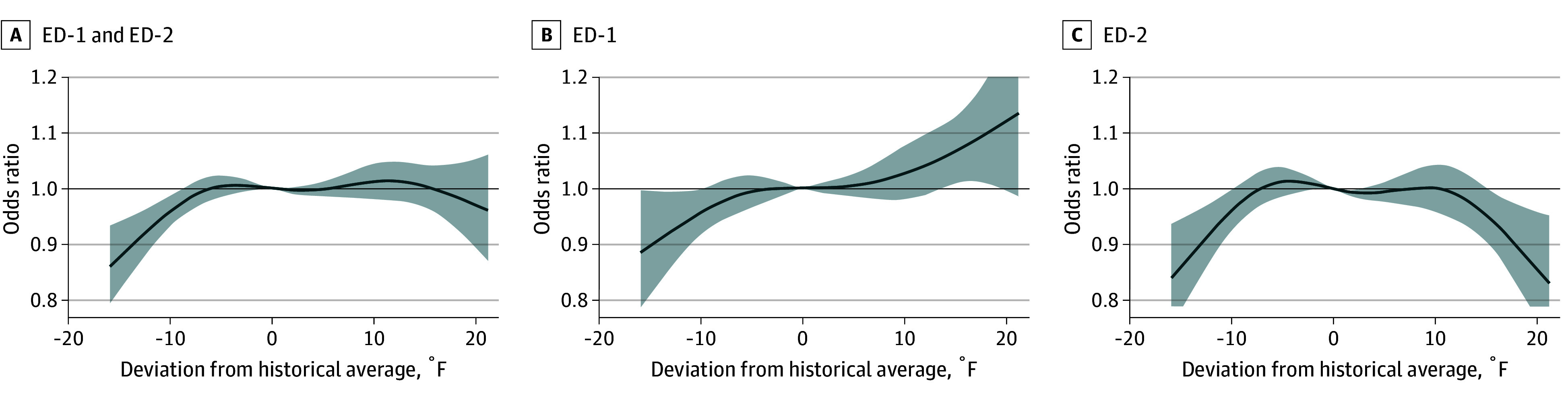
Graph of Cumulative Odds Ratios (ORs) and 95% CIs of All-Cause Emergency Department (ED) Visits From May to September 2022 to 2024, Among Adults Aged 65 Years or Older as a Function of Daily Maximum Heat Index (HI_max_) Anomalies Compared With the Reference Value 0, Representing the Absence of Anomalous HI_max_ OR is displayed on the y-axis, and HI_max_ anomalies is displayed on the x-axis. Panel A represents data from both ED sites combined, Panel B represents ED-1 only, and Panel C represents ED-2 only. Daily maximum heat index anomalies were calculated from 30-year baseline (1992-2021). Dark gray curves indicate cumulative odds, and gray shading indicates 95% CIs. ED-1 indicates EDs predominantly serving Medicaid-enrolled patients from minoritized racial and ethnic groups, and ED-2 indicates EDs predominantly serving White, privately insured patients.

At ED-1, utilization odds increased linearly with daily warm HI_max_ anomalies, reaching statistical significance on days 15 °F (OR, 1.07 [95% CI, 1.01-1.13]) to 18 °F (OR, 1.10 [95% CI, 1.01-1.20]) warmer than average and peaking on days 21 °F warmer than average (OR, 1.13 [95% CI, 0.99-1.30]) (Figure 2 and eTable 2 in Supplement 2). Of the 14 days with HI_max_ anomalies of 15 °F or more in our study, 7 (50%) did not have issued NYC heat advisories (eTable 3 in Supplement 1). At ED-2, progressively lower odds were observed on days 16 °F (OR, 0.94 [95% CI, 0.88-0.99]) to 21 °F (OR, 0.84 [95% CI, 0.73-0.95]) warmer than average.

In sensitivity analyses, exposure-response estimates were directionally consistent at ED-1 and ED-2 across alternative model specifications, including inclusion of a quadratic term, variation in spline flexibility (2 to 6 degrees of freedom) and internal knot placement (tenth, fiftieth, and ninetieth percentiles vs twenty-fifth, fiftieth, and seventy-fifth percentiles) (eFigures 3 to 8 in Supplement 1), exclusion of the 564 patients who presented to both EDs (eFigure 9 in Supplement 1), and stratification by patient demographic characteristics (eFigures 10 to 19 in Supplement 1). Stratified analyses did not alter the overall patterns observed in primary models; however, estimates within numerous strata were imprecise, with wide 95% CIs reflecting limited sample sizes.

Absolute T_max_ exposure-based models also demonstrated directionally similar exposure-response estimates at both ED sites. In T_max_ anomaly exposure-based models, increased odds of ED-1 use on warmer than average days were not observed. At ED-2, no association between T_max_ anomalies and odds of ED use was observed (eFigure 20 in Supplement 1).

## Discussion

The association between extreme heat and all-cause ED use by older adults in an urban health care setting varied by ED location. Importantly, we found increased odds of ED visits at HI_max_ below threshold values currently used for NYC heat advisory warnings. We also found higher odds of all-cause ED visits on days with anomalously high HI_max_ (15 °F or more above average), of which 50% did not trigger NYC heat advisories. Our findings suggest that health care systems are well-suited to use EHR data to guide tailored health-based heat adaptation interventions to reduce acute health care utilization.

Our findings may corroborate known heat-health inequities in NYC. ED-1 showed stronger associations between warmer HI_max_ and HI_max_ anomalies and odds of all-cause ED use compared with ED-2. Compared with ED-2, ED-1 is located in a community with a higher burden of social and structural drivers of intra-urban extreme heat vulnerability (eg, lower household income, limited green space).^21,22,23^ The NYC heat vulnerability index (HVI) scores neighborhoods based on quintiles from 1 (lowest risk) to 5 (highest risk) derived from the statistical modeling of factors determined to be highly associated with mortality during and immediately following extreme heat events, such as average surface temperature, percentage vegetation cover, percentage of households reporting air conditioning access, percentage of residents reported as non-Hispanic or Latino Black, and median household income.^24^ ED-1’s neighborhood had a higher NYC HVI score of 3, compared with ED-2’s neighborhood, which had a NYC HVI score of 1. In this way, our findings underscore the importance of future EHR-based research that pairs the identification of locally relevant exposure thresholds with systemic evaluations of how social and structural risk factors may modify those thresholds. Such research holds promise to inform tailored, health care system-based heat warning strategies to reduce preventable heat-associated acute care use.

For example, the aforementioned NYC heat advisory threshold reduction resulted in 32% less city-wide heat-related hospitalizations.^1^ Using mean attributable fraction, we roughly estimated that a heat warning system that is 32% effective triggered on days with HI_max_ of 90 °F or more and on days with HI_max_ anomalies of 15 °F or more above average without issued heat advisories may have prevented approximately 116 ED-1 visits during our study period (eTable 3 in Supplement 1).^25^

The inverse U-shaped association between anomalous HI_max_ and ED-2 use, robust across sensitivity models with varying spline flexibility and internal knot placements, is a notable finding. Existing theoretical frameworks (eg, negative affect escape theory) have been developed to explain similarly shaped associations between extreme heat and behaviorally-patterned outcomes, such as crime.^26^ ED-2 serves NYC communities with fewer social and structural drivers of heat-health vulnerability. Therefore, our findings may suggest that ED-2 patients may have greater ability to engage in heat adaptive behaviors at anomalously warm temperatures (eg, working from home, accessing air conditioning), warranting further study.

Notably, sensitivity analyses using T_max_ did not demonstrate significant associations between anomalously warm T_max_ and ED use at either study site. Prior population-scale epidemiologic studies of the impact of high temperature exposure on health have inconsistently detected associations between high levels of humidity and heat-related mortality or morbidity.^27^ In contrast, human physiological studies have consistently demonstrated that humidity compounds thermal stress, primarily by limiting evaporative heat loss, supporting the use of composite exposure metrics, such as HI_max_.^7,28,29^ Our findings may suggest that the mechanisms underlying the differences in ED use observed with anomalous heat exposure at ED-1 and ED-2 may be influenced by humidity-driven thermal stress, highlighting an important area for future research.

### Limitations

This study has limitations. Our study is limited by using HI_max_ values derived from a single ground monitor, which may have introduced the risk of exposure misclassification and ecological fallacy. However, the majority of included patients were NYC residents, and the LaGuardia Airport monitor site is highly associated with other major NYC monitors. To facilitate direct comparisons with NYC heat advisories, we did not operationalize other metrics of extreme heat (eg, wet bulb globe temperature), which may better capture thermal stress.^30^ We included all ED visits, not excluding repeat encounters, which introduced the risk of selection bias. The single urban health care system focus of our study limits generalizability. However, our findings were observed across 2 geographically distinct EDs (approximately10 miles apart) that serve demographically different patient catchment populations, suggesting a degree of generalizability across variable urban settings.

## Conclusions

Health care systems can play a crucial role in complementing municipal efforts to reduce extreme heat-driven acute health care use in at-risk populations. By leveraging robust EHR data and diverse measures of extreme heat exposure, health care systems can optimize health-based heat warning interventions to save lives and improve health during extreme heat events.

## Notes

Supplement 1. **eTable 1.** Odds of daily maximum heat index (HI_max_) associated all-cause ED visits overall, at ED-1, and ED-2 **eTable 2.** Odds of daily HI_max_ anomaly associated all-cause ED visits overall, at ED-1, and ED-2 **eTable 3.** Number of days and ED-1 visits across high HI_max_ and HI_max_ anomaly thresholds **eFigure 1.** Autocorrelative function analysis **eFigure 2.** Spearman correlation analysis of daily air quality index and all-cause ED visits **eFigure 3.** ED-1 models with HI_max_ modeled with included quadratic term and varied spline flexibility **eFigure 4.** ED-1 models with HI_max_ anomalies modeled with included quadratic term and varied spline flexibility **eFigure 5.** ED-2 models with HI_max_ modeled with included quadratic term and varied spline flexibility **eFigure 6.** ED-2 models with HI_max_ anomalies modeled with included quadratic term and varied spline flexibility **eFigure 7.** HI_max_ models with alternative internal knot placements **eFigure 8.** HI_max_ anomaly models with alternative internal knot placements **eFigure 9.** Models excluding individuals seen at both ED sites **eFigure 10. **HI_max_ models stratified by patient sex **eFigure 11.** HI_max_ anomaly models stratified by patient sex **eFigure 12.** HI_max_ models stratified by patient age **eFigure 13.** HI_max_ anomaly models stratified by patient age **eFigure 14.** ED-1 models as a function of HI_max_ stratified by patient insurance **eFigure 15.** ED-1 models as a function of HI_max_ anomalies stratified by patient insurance **eFigure 16.** ED-2 models as a function of HI_max_ stratified by patient insurance **eFigure 17.** ED-2 models as a function of HI_max_ anomalies stratified by patient insurance **eFigure 18.** HI_max_ models stratified by patient self-reported race/ ethnicity **eFigure 19.** HI_max_ anomaly models stratified by patient self-reported race/ ethnicity **eFigure 20.** Models with substituted daily maximum ambient temperature (T_max_) as the exposure

Supplement 2.
Data Sharing Statement
