# Supplementary material for: Extreme Urban Heat and Emergency Department Visits in Older Adults
Source: JAMA Netw Open. 2026 Mar 20;9(3):e262645. doi: 10.1001/jamanetworkopen.2026.2645 (PMC13005158; doi:10.1001/jamanetworkopen.2026.2645)

Supplemental Online Content

Siau E, Silva GS, Lu J, et al. Extreme urban heat and emergency department visits in older adults. *JAMA Netw Open*. 2026;9(3):e262645. doi:10.1001/jamanetworkopen.2026.2645

eTable 1: Odds of daily maximum heat index (HI_{max}) associated all-cause ED visits overall, at ED-1, and ED-2
eTable 2: Odds of daily HI_{max} anomaly associated all-cause ED visits overall, at ED-1, and ED-2
eTable 3: Number of days and ED-1 visits across high HI_{max} and HI_{max} anomaly thresholds
eFigure 1: Autocorrelative function analysis
eFigure 2: Spearman correlation analysis of daily air quality index and all-cause ED visits
eFigure 3: ED-1 models with HI_{max} modeled with included quadratic term and varied spline flexibility
eFigure 4: ED-1 models with HI_{max} anomalies modeled with included quadratic term and varied spline flexibility
eFigure 5: ED-2 models with HI_{max} modeled with included quadratic term and varied spline flexibility
eFigure 6: ED-2 models with HI_{max} anomalies modeled with included quadratic term and varied spline flexibility
eFigure 7: HI_{max} models with alternative internal knot placements
eFigure 8: HI_{max} anomaly models with alternative internal knot placements
eFigure 9: Models excluding individuals seen at both ED sites
eFigure 10: HI_{max} models stratified by patient sex
eFigure 11: HI_{max} anomaly models stratified by patient sex
eFigure 12: HI_{max} models stratified by patient age
eFigure 13: HI_{max} anomaly models stratified by patient age
eFigure 14: ED-1 models as a function of HI_{max} stratified by patient insurance
eFigure 15: ED-1 models as a function of HI_{max} anomalies stratified by patient insurance
eFigure 16: ED-2 models as a function of HI_{max} stratified by patient insurance
eFigure 17: ED-2 models as a function of HI_{max} anomalies stratified by patient insurance
eFigure 18: HI_{max} models stratified by patient self-reported race/ ethnicity
eFigure 19: HI_{max} anomaly models stratified by patient self-reported race/ ethnicity
eFigure 20: Models with substituted daily maximum ambient temperature (T_{max}) as the exposure

This supplemental material has been provided by the authors to give readers additional information about their work.

© 2026 Siau E et al. *JAMA Network Open*.

eTable 1. Cumulative odds and 95% confidence intervals of all-cause ED visits overall, at ED-1, and ED-2 NYU, between May to September 2022-2024, among adults 65 years and older as a function of daily maximum heat index.

Maximum Heat Index	Overall	ED-1	ED-2
57°F	1.01 (1.00, 1.01)	1.01 (1.00, 1.02)	1.00 (0.99, 1.01)
58°F	1.01 (0.99, 1.03)	1.02 (1.00, 1.05)	1.00 (0.98, 1.03)
59°F	1.02 (0.99, 1.04)	1.04 (1.00, 1.08)	1.00 (0.97, 1.04)
60°F	1.02 (0.99, 1.06)	1.05 (1.00, 1.10)	1.00 (0.96, 1.05)
61°F	1.03 (0.99, 1.07)	1.06 (1.00, 1.13)	1.01 (0.95, 1.06)
62°F	1.04 (0.99, 1.09)	1.07 (1.00, 1.15)	1.01 (0.95, 1.08)
63°F	1.04 (0.99, 1.10)	1.08 (1.00, 1.17)	1.01 (0.94, 1.09)
64°F	1.05 (0.99, 1.11)	1.09 (1.00, 1.19)	1.02 (0.94, 1.10)
65°F	1.05 (0.99, 1.12)	1.10 (1.00, 1.20)	1.02 (0.94, 1.10)
66°F	1.06 (0.99, 1.13)	1.10 (1.01, 1.21)	1.02 (0.94, 1.11)
67°F	1.06 (1.00, 1.13)	1.11 (1.01, 1.22)	1.03 (0.95, 1.12)
68°F	1.07 (1.00, 1.14)	1.12 (1.01, 1.23)	1.03 (0.95, 1.13)
69°F	1.07 (1.01, 1.14)	1.12 (1.02, 1.23)	1.04 (0.96, 1.13)
70°F	1.08 (1.01, 1.15)	1.12 (1.02, 1.24)	1.05 (0.96, 1.14)
71°F	1.08 (1.02, 1.15)	1.13 (1.02, 1.24)	1.05 (0.97, 1.14)
72°F	1.09 (1.02, 1.16)	1.13 (1.03, 1.24)	1.06 (0.97, 1.15)
73°F	1.09 (1.03, 1.16)	1.13 (1.03, 1.24)	1.06 (0.98, 1.15)
74°F	1.09 (1.03, 1.16)	1.13 (1.03, 1.24)	1.06 (0.98, 1.15)
75°F	1.09 (1.03, 1.16)	1.13 (1.03, 1.24)	1.07 (0.98, 1.15)
76°F	1.10 (1.03, 1.16)	1.13 (1.03, 1.24)	1.07 (0.99, 1.16)
77°F	1.10 (1.03, 1.16)	1.13 (1.03, 1.24)	1.07 (0.99, 1.16)
78°F	1.10 (1.03, 1.16)	1.13 (1.03, 1.24)	1.07 (0.99, 1.16)
79°F	1.09 (1.03, 1.16)	1.13 (1.03, 1.24)	1.07 (0.99, 1.15)
80°F	1.09 (1.03, 1.16)	1.13 (1.03, 1.24)	1.06 (0.98, 1.15)
81°F	1.09 (1.03, 1.16)	1.13 (1.03, 1.24)	1.06 (0.98, 1.15)
82°F	1.09 (1.03, 1.16)	1.13 (1.03, 1.24)	1.06 (0.98, 1.15)
83°F	1.09 (1.02, 1.15)	1.13 (1.03, 1.24)	1.05 (0.97, 1.14)
84°F	1.09 (1.02, 1.15)	1.13 (1.03, 1.24)	1.05 (0.97, 1.14)
85°F	1.08 (1.02, 1.15)	1.13 (1.03, 1.24)	1.05 (0.96, 1.13)
86°F	1.08 (1.02, 1.15)	1.13 (1.03, 1.25)	1.04 (0.96, 1.13)
87°F	1.08 (1.01, 1.15)	1.14 (1.03, 1.25)	1.04 (0.95, 1.13)
88°F	1.08 (1.01, 1.15)	1.14 (1.03, 1.26)	1.03 (0.95, 1.12)
89°F	1.08 (1.01, 1.15)	1.14 (1.04, 1.26)	1.03 (0.94, 1.12)
90°F	1.08 (1.01, 1.15)	1.15 (1.04, 1.27)	1.02 (0.94, 1.12)
91°F	1.08 (1.01, 1.15)	1.15 (1.05, 1.27)	1.02 (0.93, 1.11)
92°F	1.08 (1.01, 1.15)	1.16 (1.05, 1.28)	1.02 (0.93, 1.11)
93°F	1.08 (1.01, 1.15)	1.17 (1.06, 1.29)	1.02 (0.93, 1.11)
94°F	1.08 (1.01, 1.16)	1.18 (1.07, 1.30)	1.01 (0.93, 1.11)
95°F	1.09 (1.02, 1.16)	1.19 (1.07, 1.31)	1.01 (0.93, 1.10)
96°F	1.09 (1.02, 1.16)	1.19 (1.08, 1.32)	1.01 (0.93, 1.10)
97°F	1.09 (1.02, 1.17)	1.20 (1.09, 1.33)	1.01 (0.92, 1.10)
98°F	1.10 (1.02, 1.17)	1.21 (1.10, 1.34)	1.01 (0.92, 1.11)
99°F	1.10 (1.03, 1.18)	1.23 (1.10, 1.36)	1.01 (0.92, 1.11)
100°F	1.10 (1.03, 1.19)	1.24 (1.11, 1.38)	1.01 (0.92, 1.11)
101°F	1.11 (1.03, 1.19)	1.24 (1.11, 1.39)	1.01 (0.91, 1.11)

© 2026 Siau E et al. *JAMA Network Open*.

eTable 2. Cumulative odds and 95% confidence intervals of all-cause ED visits overall, at ED-1 and ED-2, between May to September 2022-2024, among adults 65 years and older by daily maximum heat index anomalies.[a]

Maximum Heat Index Anomaly	Overall	ED-1	ED-2
-16°F	0.86 (0.79, 0.93)	0.89 (0.78, 1.00)	0.84 (0.75, 0.94)
-15°F	0.88 (0.82, 0.94)	0.90 (0.81, 1.00)	0.86 (0.78, 0.95)
-14°F	0.89 (0.84, 0.95)	0.91 (0.83, 1.00)	0.88 (0.81, 0.95)
-13°F	0.91 (0.87, 0.96)	0.92 (0.86, 0.99)	0.90 (0.84, 0.96)
-12°F	0.93 (0.89, 0.97)	0.93 (0.88, 0.99)	0.92 (0.87, 0.98)
-11°F	0.94 (0.91, 0.98)	0.95 (0.90, 1.00)	0.94 (0.90, 0.99)
-10°F	0.96 (0.93, 0.99)	0.96 (0.92, 1.00)	0.96 (0.92, 1.00)
-9°F	0.97 (0.95, 1.00)	0.97 (0.93, 1.01)	0.98 (0.94, 1.01)
-8°F	0.98 (0.96, 1.01)	0.98 (0.94, 1.01)	0.99 (0.96, 1.02)
-7°F	0.99 (0.97, 1.02)	0.98 (0.95, 1.02)	1.00 (0.97, 1.03)
-6°F	1.00 (0.98, 1.02)	0.99 (0.96, 1.02)	1.01 (0.98, 1.04)
-5°F	1.00 (0.98, 1.02)	0.99 (0.97, 1.02)	1.01 (0.99, 1.04)
-4°F	1.01 (0.99, 1.02)	1.00 (0.97, 1.02)	1.01 (0.99, 1.04)
-3°F	1.01 (0.99, 1.02)	1.00 (0.98, 1.02)	1.01 (0.99, 1.03)
-2°F	1.00 (1.00, 1.01)	1.00 (0.99, 1.01)	1.01 (1.00, 1.02)
-1°F	1.00 (1.00, 1.01)	1.00 (0.99, 1.01)	1.00 (1.00, 1.01)
1°F	1.00 (0.99, 1.00)	1.00 (1.00, 1.01)	1.00 (0.99, 1.00)
2°F	1.00 (0.99, 1.00)	1.00 (0.99, 1.01)	0.99 (0.98, 1.00)
3°F	1.00 (0.99, 1.01)	1.00 (0.99, 1.02)	0.99 (0.98, 1.00)
4°F	1.00 (0.99, 1.01)	1.00 (0.99, 1.02)	0.99 (0.98, 1.01)
5°F	1.00 (0.99, 1.01)	1.01 (0.99, 1.03)	0.99 (0.98, 1.01)
6°F	1.00 (0.98, 1.02)	1.01 (0.98, 1.03)	1.00 (0.97, 1.02)
7°F	1.00 (0.98, 1.03)	1.01 (0.98, 1.04)	1.00 (0.97, 1.03)
8°F	1.01 (0.98, 1.03)	1.02 (0.98, 1.05)	1.00 (0.97, 1.03)
9°F	1.01 (0.98, 1.04)	1.02 (0.98, 1.07)	1.00 (0.96, 1.04)
10°F	1.01 (0.98, 1.04)	1.03 (0.98, 1.08)	1.00 (0.96, 1.04)
11°F	1.01 (0.98, 1.05)	1.03 (0.98, 1.09)	1.00 (0.95, 1.04)
12°F	1.01 (0.98, 1.05)	1.04 (0.99, 1.10)	0.99 (0.94, 1.04)
13°F	1.01 (0.98, 1.05)	1.05 (1.00, 1.11)	0.98 (0.93, 1.03)
14°F	1.01 (0.97, 1.04)	1.06 (1.00, 1.12)	0.97 (0.92, 1.02)
15°F	1.00 (0.97, 1.04)	1.07 (1.01, 1.13)	0.95 (0.91, 1.00)
16°F	1.00 (0.96, 1.04)	1.08 (1.01, 1.15)	0.94 (0.88, 0.99)
17°F	0.99 (0.95, 1.04)	1.09 (1.01, 1.17)	0.92 (0.86, 0.98)
18°F	0.99 (0.93, 1.04)	1.10 (1.01, 1.20)	0.90 (0.83, 0.97)
19°F	0.98 (0.91, 1.05)	1.11 (1.00, 1.23)	0.88 (0.80, 0.97)
20°F	0.97 (0.89, 1.05)	1.12 (1.00, 1.26)	0.86 (0.76, 0.96)
21°F	0.96 (0.88, 1.06)	1.13 (0.99, 1.30)	0.84 (0.73, 0.95)

a. Daily maximum heat index anomalies were calculated from 30-year baseline (1992-2021).

© 2026 Siau E et al. *JAMA Network Open*.

eTable 3. Number of days and associated daily mean and median ED-1 visits within our study period observed across various high heat index and heat index anomaly thresholds.[a]

Heat Category	Days	ED-1 Visit	
		Mean (SD)	Median (IQR)
**Day with HI_{max} and**			
Heat Advisory	25	57.1 (8.5)	55.0 (51.0, 62.0)
95°F+ (no Heat Advisory)	6	56.2 (7.6)	57.5 (52.3, 60.0)
90°F -94°F	40	55.5 (8.4)	54.0 (49.8, 61.3)
85°F -89°F	65	53.6 (10.0)	53.0 (45.0, 61.0)
80°F -84°F	124	53.1 (8.0)	53.0 (47.0, 55.3)
<80°F	199	52.8 (8.9)	53.0 (46.0, 58.5)
**Day with HI_{max} Anomaly ≥15°F and**			
Heat Advisory	7	61.0 (9.9)	61.5 (56.5, 65.8)
95°F+ (no Heat Advisory)	3	53.5 (3.0)	53.0 (51.0, 55.5)
90°F -94°F	2	56.0 (18.4)	56.0 (49.5, 62.5)
< 90°F (both are 81°F and 84°F)	2	51.0 (12.7)	51.0 (46.5, 55.5)

a. A Heat Advisory represents two consecutive days with recorded maximum heat index values 95°F or higher or days with recorded heat index values 100°F or higher for any period of time.

© 2026 Siau E et al. *JAMA Network Open*.

eFigure 1. Autocorrelative function analysis demonstrating no significant correlation between lagged days and daily maximum heat index and all-cause emergency department visit associations during summer months (May to September).

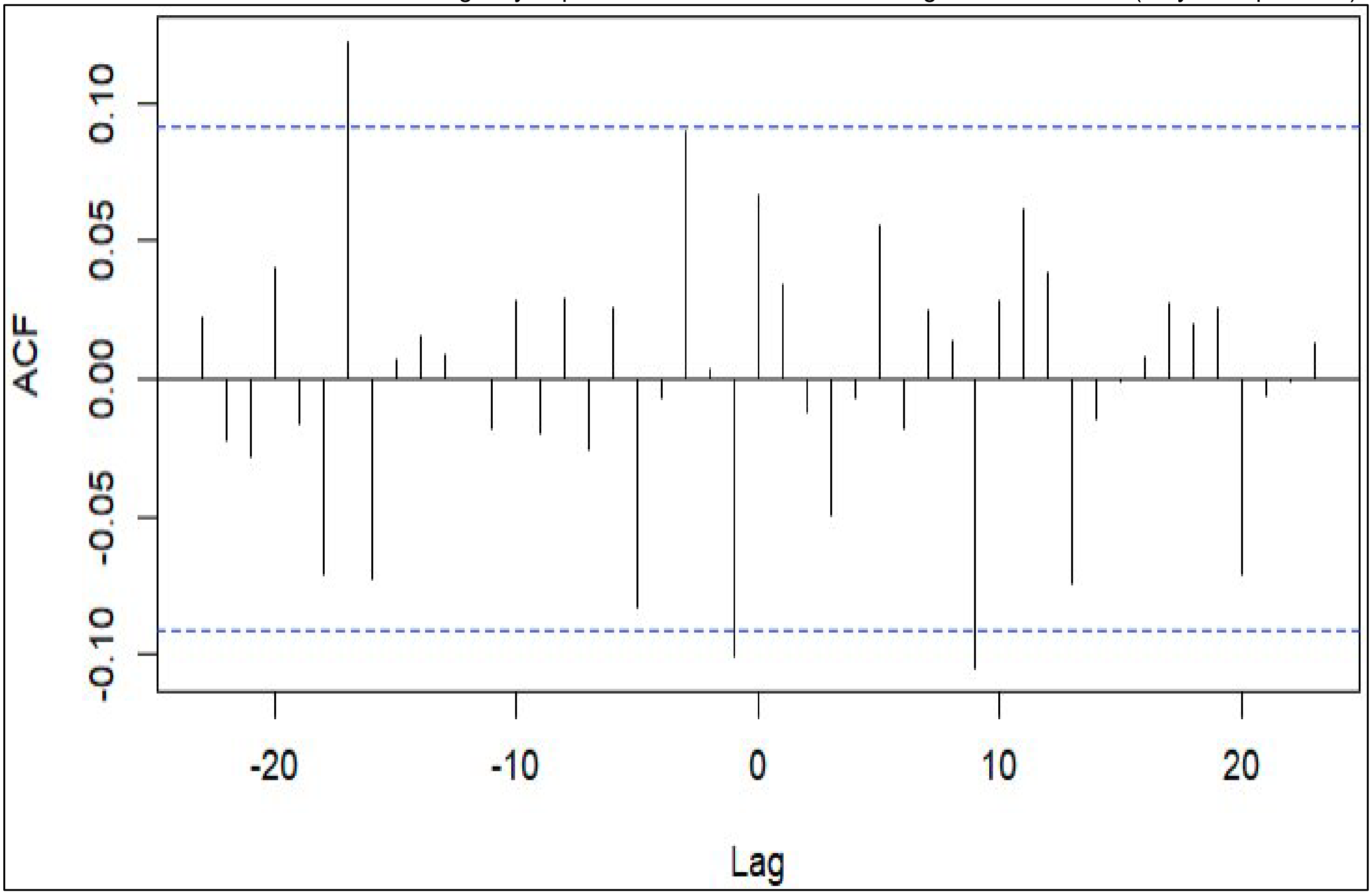

© 2026 Siau E et al. *JAMA Network Open*.

eFigure 2. Spearman correlation coefficients reported for comparisons between daily air quality index (AQI) overall and pollutant-specific values and daily all-cause emergency department visits.[a]

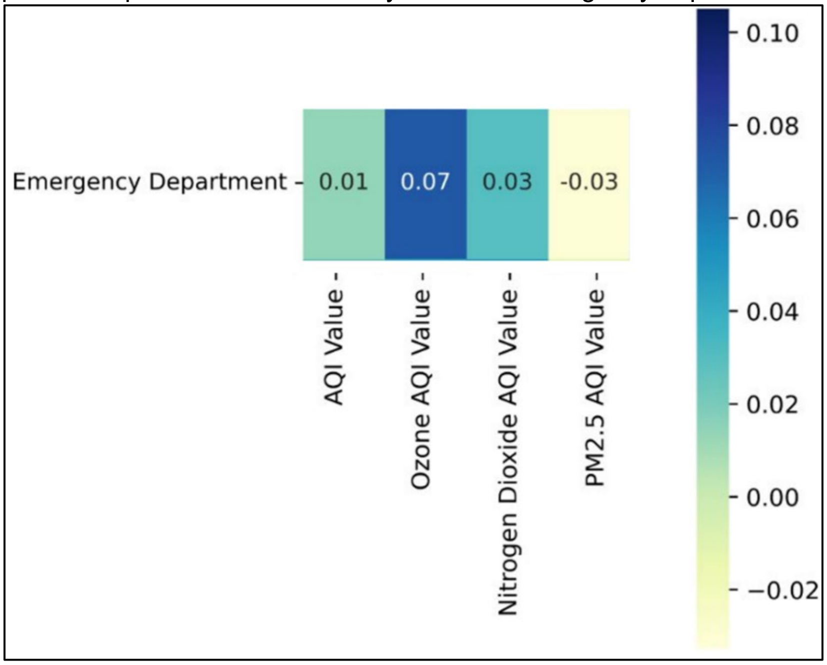

a. AQI values were obtained from Queens College monitor-derived data through the United States Environmental Protection Agency AirNow database.

© 2026 Siau E et al. *JAMA Network Open*.

eFigure 3. Graph of cumulative odds (red curve) and 95% confidence intervals (gray margin) of all-cause ED-1 visits from May to September 2022-2024, among adults 65 years or older as a function of daily maximum heat index (HI_{max}) compared to the reference lowest HI_{max} of minimum effect (56.1°F). Odds ratio (OR) is displayed on the y-axis, and HI_{max} is displayed on the x-axis. Panel A shows models including a quadratic term for HI_{max}. Panels B–E show models in which HI_{max} was modeled using natural cubic splines with increasing flexibility: 2 degrees of freedom (Panel B), 3 degrees of freedom (Panel C), 5 degrees of freedom (Panel D), and 6 degrees of freedom (Panel E).

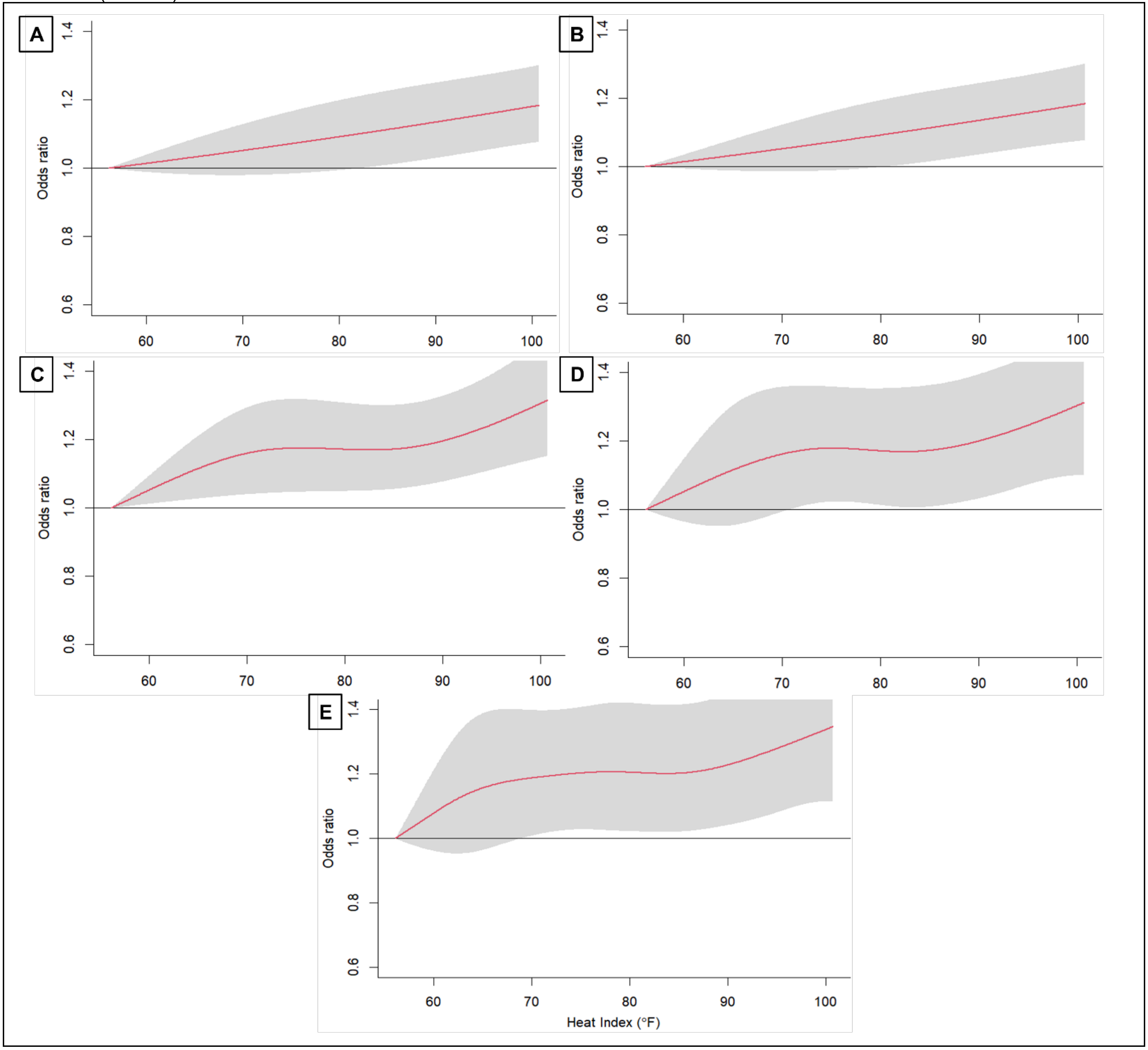

© 2026 Siau E et al. *JAMA Network Open*.

eFigure 4. Graph of cumulative odds (red curve) and 95% confidence intervals (gray margin) of all-cause ED-1 visits from May to September 2022-2024, among adults 65 years or older as a function of daily maximum heat index (HI_{max}) anomalies compared the reference value 0, representing the absence of anomalous HI_{max}. Odds ratio (OR) is displayed on the y-axis, and HI_{max} anomalies are displayed on the x-axis. Panel A shows models including a quadratic term for HI_{max}. Panels B–E show models in which HI_{max} was modeled using natural cubic splines with increasing flexibility: 2 degrees of freedom (Panel B), 3 degrees of freedom (Panel C), 5 degrees of freedom (Panel D), and 6 degrees of freedom (Panel E).

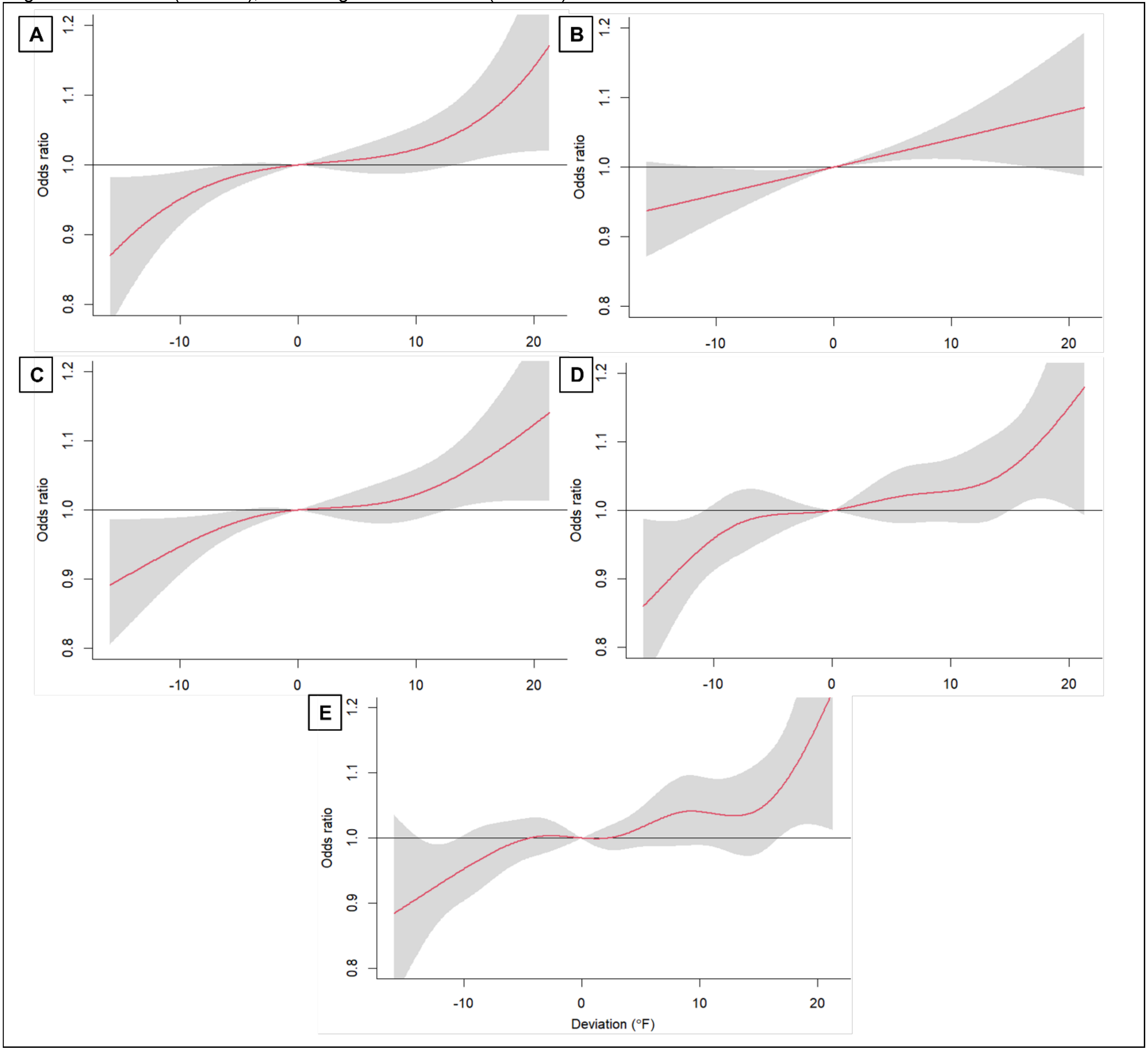

© 2026 Siau E et al. *JAMA Network Open*.

eFigure 5. Graph of cumulative odds (red curve) and 95% confidence intervals (gray margin) of all-cause ED-2 visits from May to September 2022-2024, among adults 65 years or older as a function of daily maximum heat index (HI_{max}) compared to the reference lowest HI_{max} of minimum effect (56.1°F). Odds ratio (OR) is displayed on the y-axis, and HI_{max} is displayed on the x-axis. Panel A shows models including a quadratic term for HI_{max}. Panels B–E show models in which HI_{max} was modeled using natural cubic splines with increasing flexibility: 2 degrees of freedom (Panel B), 3 degrees of freedom (Panel C), 5 degrees of freedom (Panel D), and 6 degrees of freedom (Panel E).

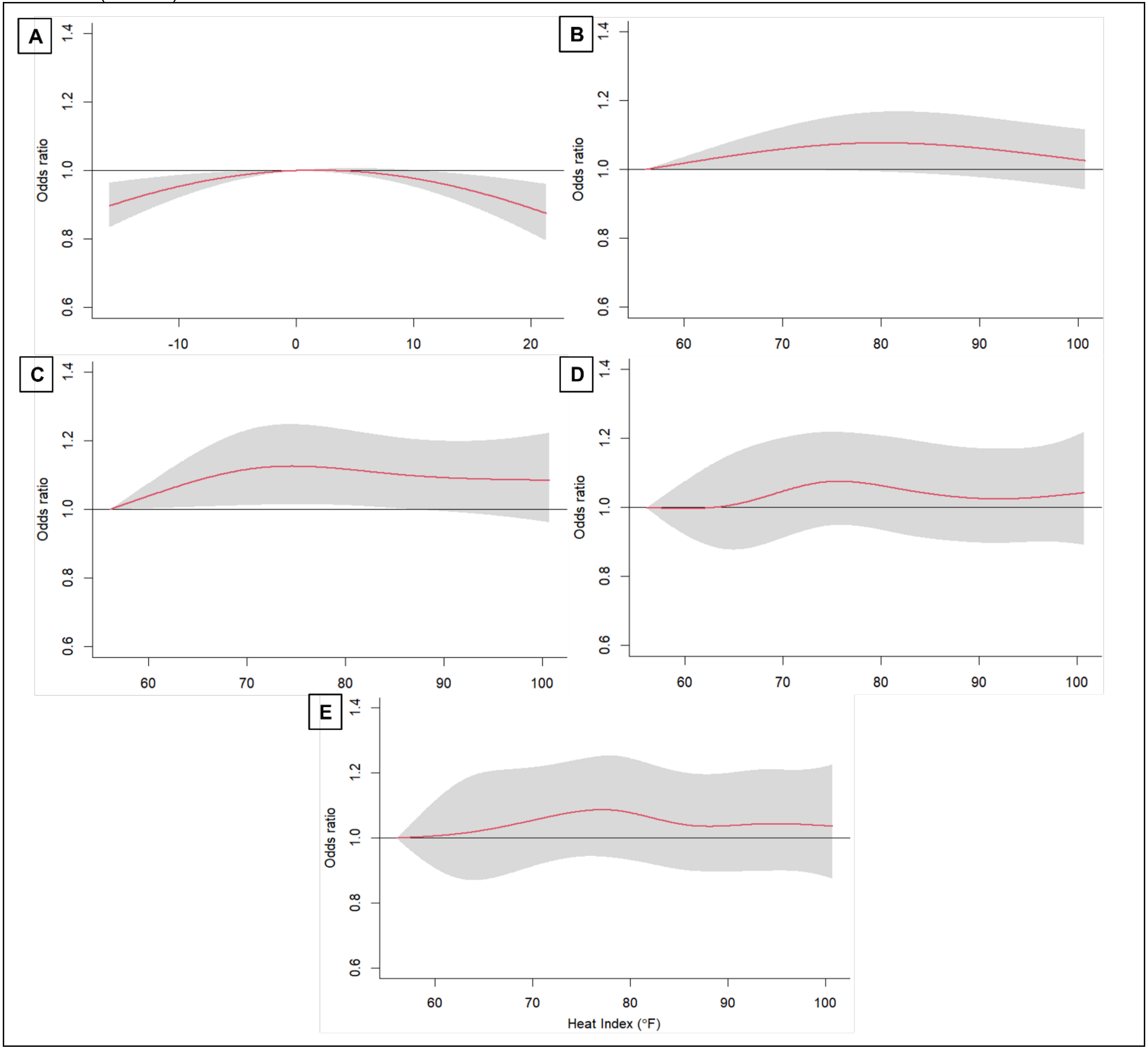

© 2026 Siau E et al. *JAMA Network Open*.

eFigure 6. Graph of cumulative odds (red curve) and 95% confidence intervals (gray margin) of all-cause ED-2 visits from May to September 2022-2024, among adults 65 years or older as a function of daily maximum heat index (HI_{max}) anomalies compared the reference value 0, representing the absence of anomalous HI_{max}. Odds ratio (OR) is displayed on the y-axis, and HI_{max} anomalies are displayed on the x-axis. Panel A shows models including a quadratic term for HI_{max}. Panels B–E show models in which HI_{max} was modeled using natural cubic splines with increasing flexibility: 2 degrees of freedom (Panel B), 3 degrees of freedom (Panel C), 5 degrees of freedom (Panel D), and 6 degrees of freedom (Panel E).

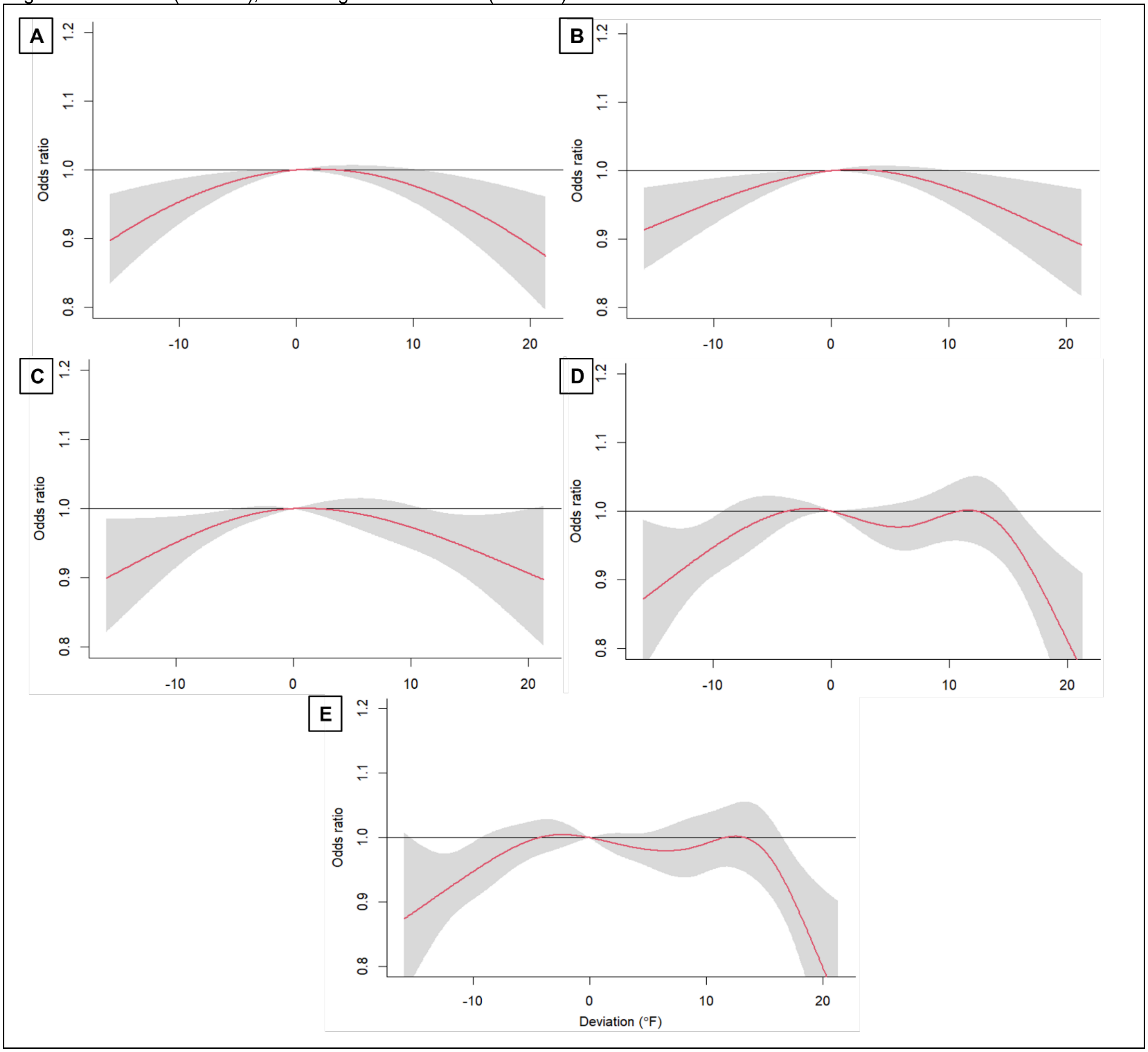

© 2026 Siau E et al. *JAMA Network Open*.

eFigure 7. Graph of cumulative odds (red curve) and 95% confidence intervals (gray margin) of all-cause ED visits from May to September 2022-2024, among adults 65 years or older as a function of daily maximum heat index (HI_{max}) compared to the reference lowest HI_{max} of minimum effect (56.1°F). Odds ratio (OR) is displayed on the y-axis, and HI_{max} is displayed on the x-axis. Panels A and B show ED-1-based models with internal knots placed at the 10th/50th/90th percentiles (Panel A) and 25th/50th/75th percentiles (Panel B), respectively. Panels C and D show ED-2-based models with the same knot placements: 10th/50th/90th percentiles (Panel C), 25th/50th/75th percentiles (Panel D).

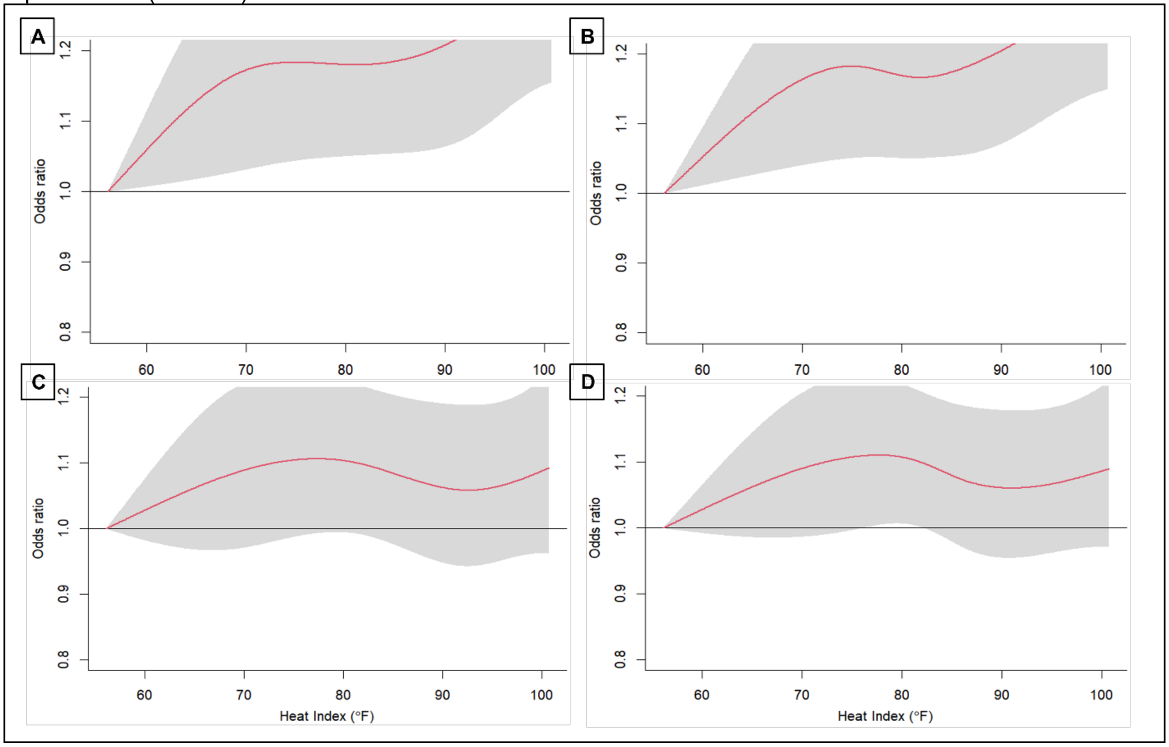

© 2026 Siau E et al. *JAMA Network Open*.

eFigure 8. Graph of cumulative odds (red curve) and 95% confidence intervals (gray margin) of all-cause ED visits from May to September 2022-2024, among adults 65 years or older as a function of daily maximum heat index (HI_{max}) anomalies compared the reference value 0, representing the absence of anomalous HI_{max}. Odds ratio (OR) is displayed on the y-axis, and HI_{max} anomalies are displayed on the x-axis. Panels A and B show ED-1-based models with internal knots placed at the 10th/50th/90th percentiles (Panel A) and 25th/50th/75th percentiles (Panel B), respectively. Panels C and D show ED-2-based models with the same knot placements: 10th/50th/90th percentiles (Panel C), 25th/50th/75th percentiles (Panel D)

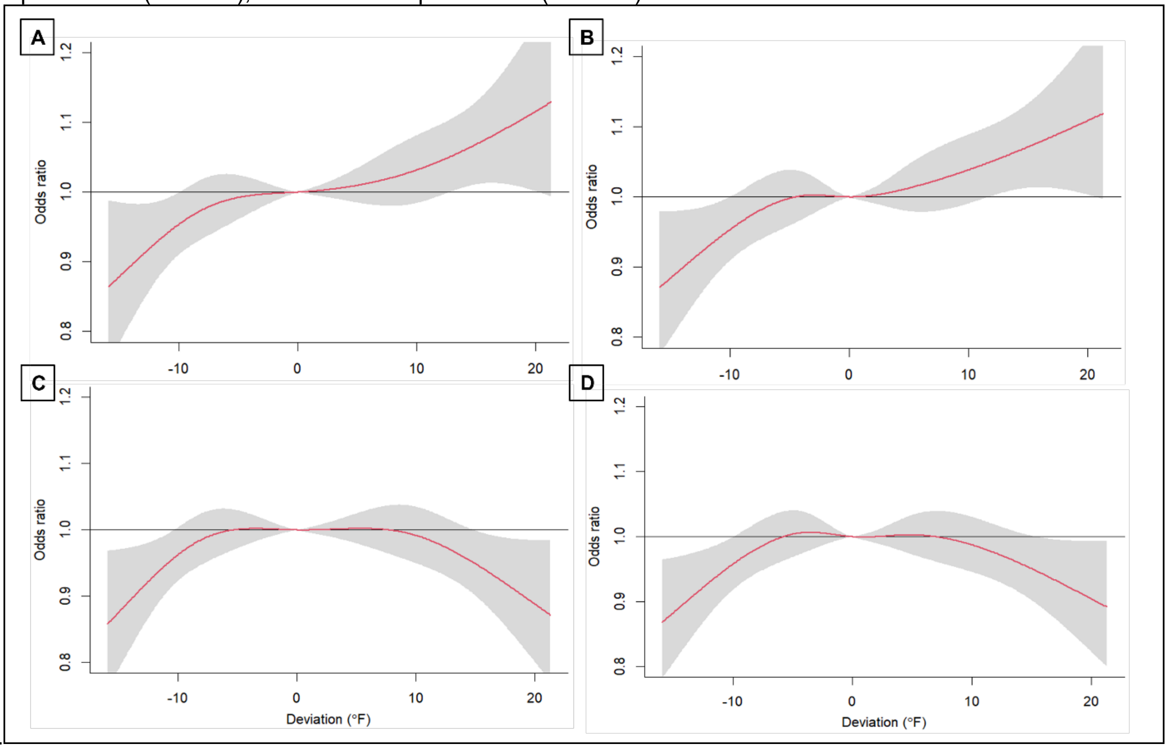

© 2026 Siau E et al. *JAMA Network Open*.

eFigure 9. Graph of cumulative odds (red curve) and 95% confidence intervals (gray margin) for all-cause emergency department (ED) visits from May to September 2022-2024, among adults 65 years or older, excluding individuals seen at both ED sites during the study period. The odds ratio (OR) is displayed on the y-axis, and daily maximum head index (HI_{max}) or HI_{max} anomalies are displayed on the x-axis. Panels A and B show ED-1-based models as a function of HI_{max} compared to the reference lowest HI_{max} of minimum effect (56.1°F) (Panel A) and HI_{max} anomalies compared the reference value 0, representing the absence of anomalous HI_{max} (Panel B). Panels C and D show ED-2-based models as a function of HI_{max} (Panel C) and HI_{max} anomalies (Panel D).

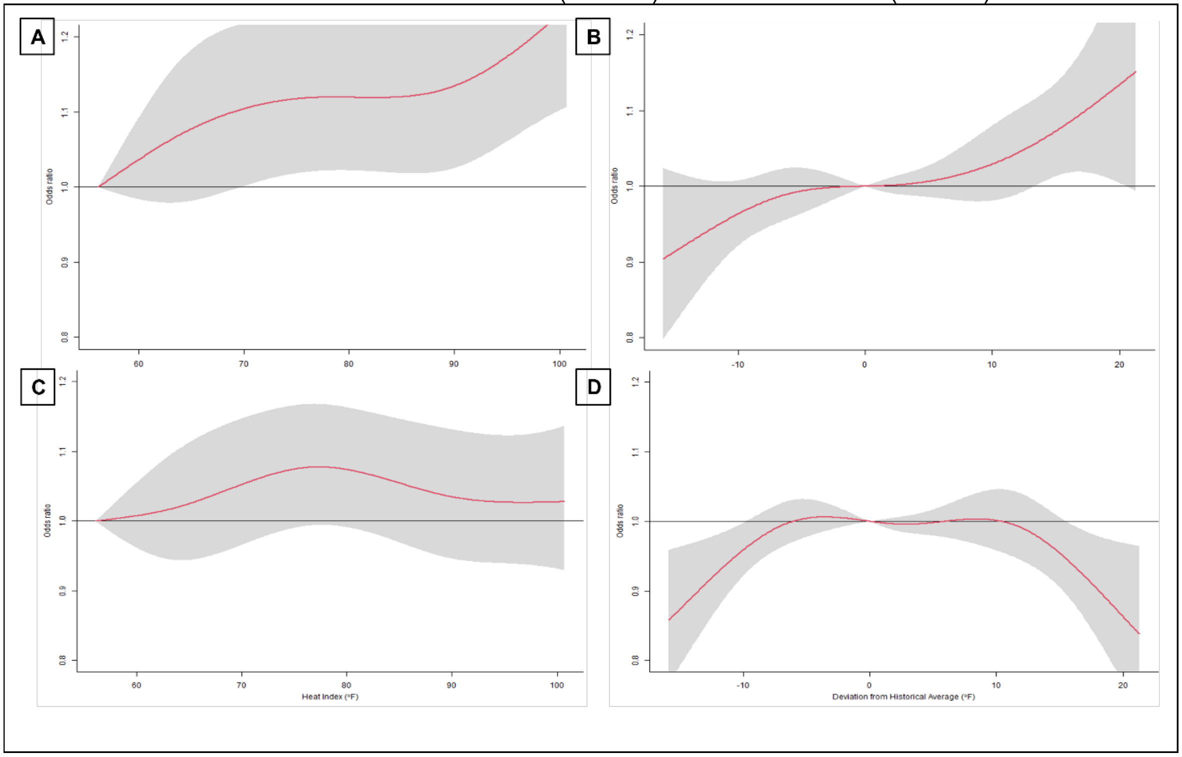

© 2026 Siau E et al. *JAMA Network Open*.

eFigure 10. Graph of cumulative odds (red curve) and 95% confidence intervals (gray margin) of all-cause ED visits from May to September 2022-2024, among adults 65 years or older as a function of daily maximum heat index (HI_{max}) compared to the reference lowest HI_{max} of minimum effect (56.1°F). Odds ratio (OR) is displayed on the y-axis, and HI_{max} is displayed on the x-axis. Panels A and B show ED-1-based models stratified by sex: male (Panel A) and female (Panel B). Panels C and D show ED-2-based models stratified by sex: male (Panel C) and female (Panel D).

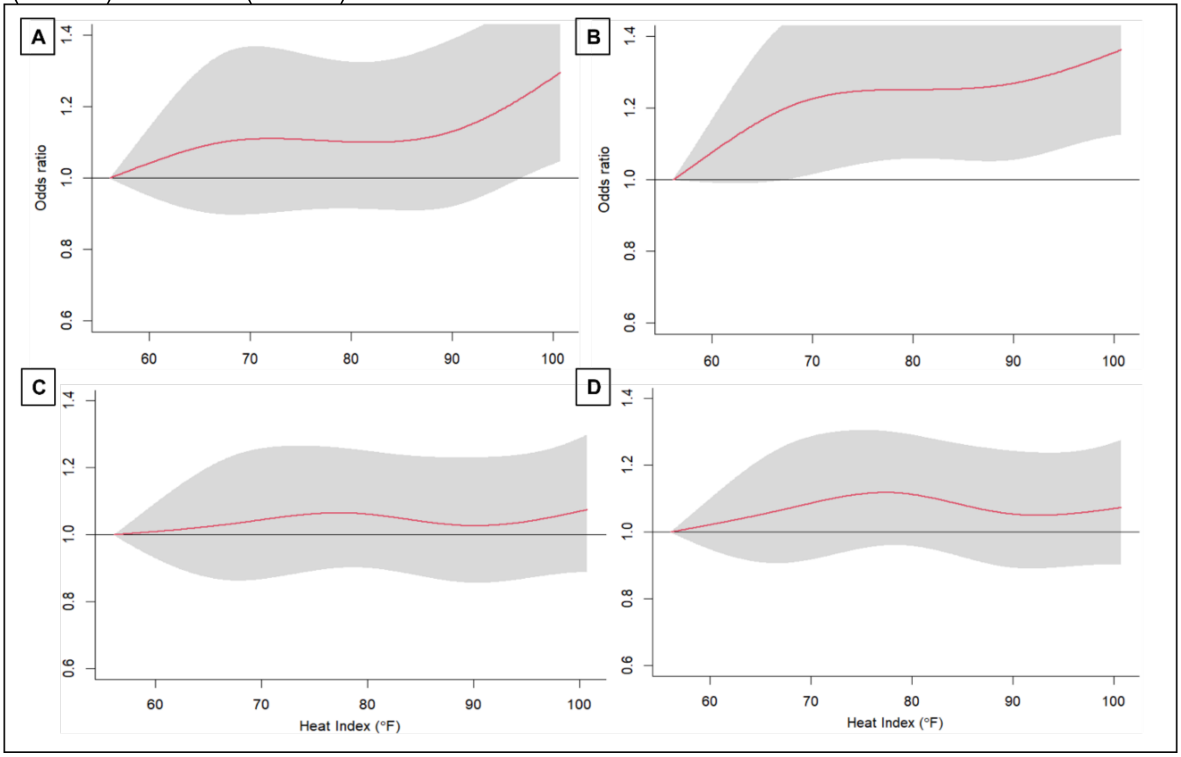

© 2026 Siau E et al. *JAMA Network Open*.

eFigure 11. Graph of cumulative odds (red curve) and 95% confidence intervals (gray margin) of all-cause ED visits from May to September 2022-2024, among adults 65 years or older as a function of daily maximum heat index (HI_{max}) anomalies compared the reference value 0, representing the absence of anomalous HI_{max}. Odds ratio (OR) is displayed on the y-axis, and daily HI_{max} anomalies are displayed on the x-axis. Panels A and B show ED-1-based models stratified by sex: male (Panel A) and female (Panel B). Panels C and D show ED-2-based models stratified by sex: male (Panel C) and female (Panel D).

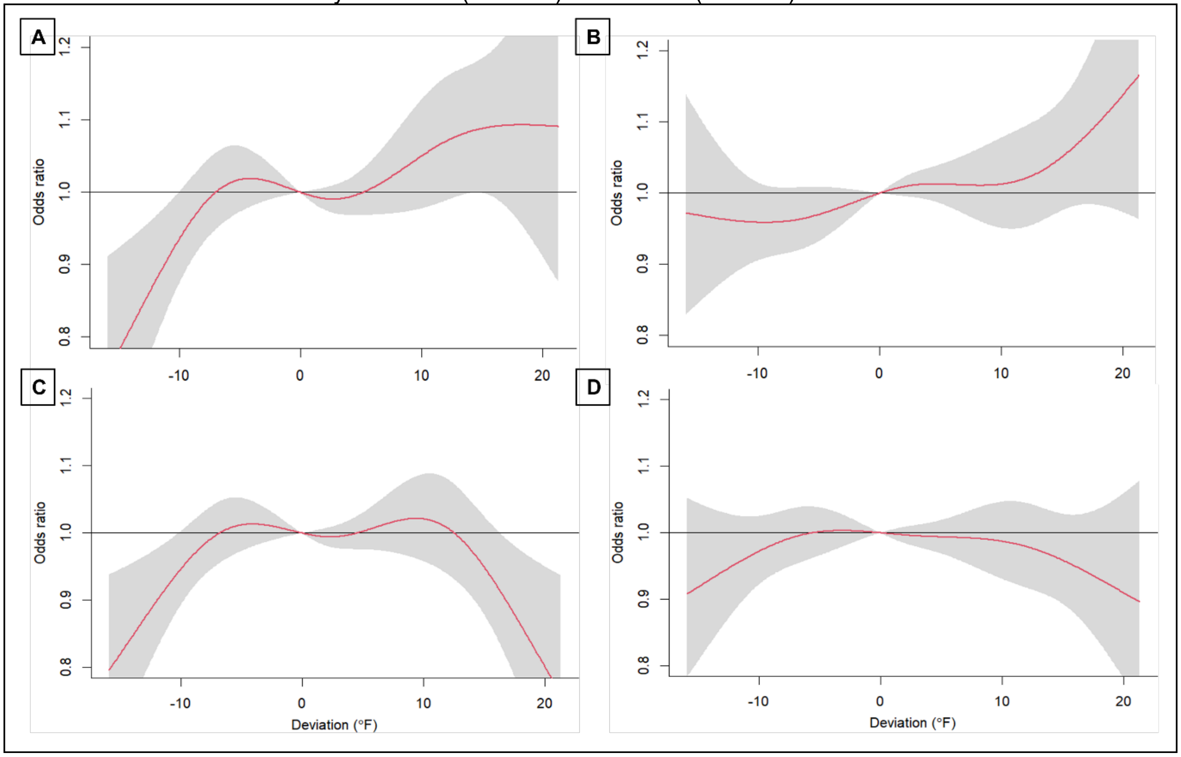

© 2026 Siau E et al. *JAMA Network Open*.

eFigure 12. Graph of cumulative odds (red curve) and 95% confidence intervals (gray margin) of all-cause ED visits from May to September 2022-2024, among adults 65 years or older as a function of daily maximum heat index (HI_{max}) compared to the reference lowest HI_{max} of minimum effect (56.1°F). Odds ratio (OR) is displayed on the y-axis, and HI_{max} is displayed on the x-axis. Panels A and B show ED-1-based models stratified by age: 65-84 years old (Panel A) and 85 years and older (Panel B). Panels C and D show ED-2-based models stratified by age: 65-84 years old (Panel C) and 85 years and older (Panel D).

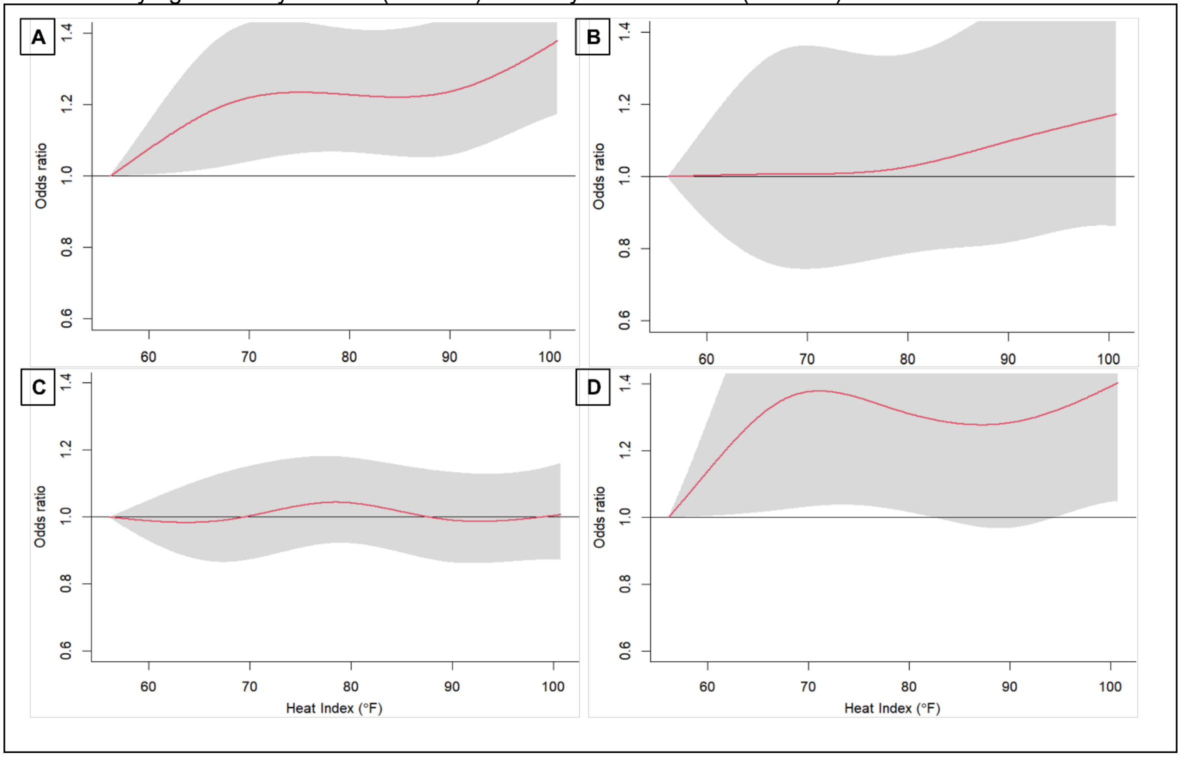

eFigure 13. Graph of cumulative odds (red curve) and 95% confidence intervals (gray margin) of all-cause ED visits from May to September 2022-2024, among adults 65 years or older as a function of daily maximum heat index (HI_{max}) anomalies compared the reference value 0, representing the absence of anomalous HI_{max}. Odds ratio (OR) is displayed on the y-axis, and HI_{max} anomalies are displayed on the x-axis. Panels A and B show ED-1-based models stratified by age: 65-84 years old (Panel A) and 85 years and older (Panel B). Panels C and D show ED-2-based models stratified by age: 65-84 years old (Panel C) and 85 years and older (Panel D).

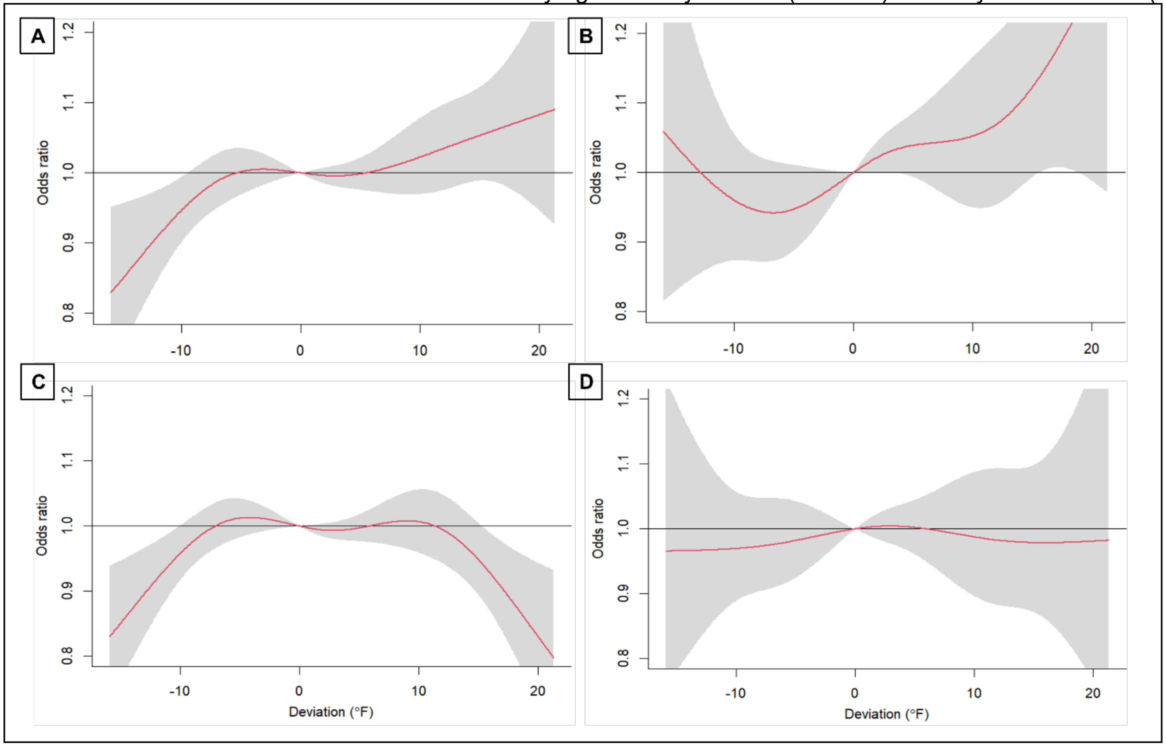

eFigure 14. Graph of cumulative odds (red curve) and 95% confidence intervals (gray margin) of all-cause ED-1 visits from May to September 2022-2024, among adults 65 years or older as a function of daily maximum heat index (HI_{max}) compared to the reference lowest HI_{max} of minimum effect (56.1°F). Odds ratio (OR) is displayed on the y-axis, and HI_{max} is displayed on the x-axis. Panels show models stratified by insurance type: private insurance (Panel A), Medicare (Panel B), Medicaid (Panel C), and missing insurance (Panel D).

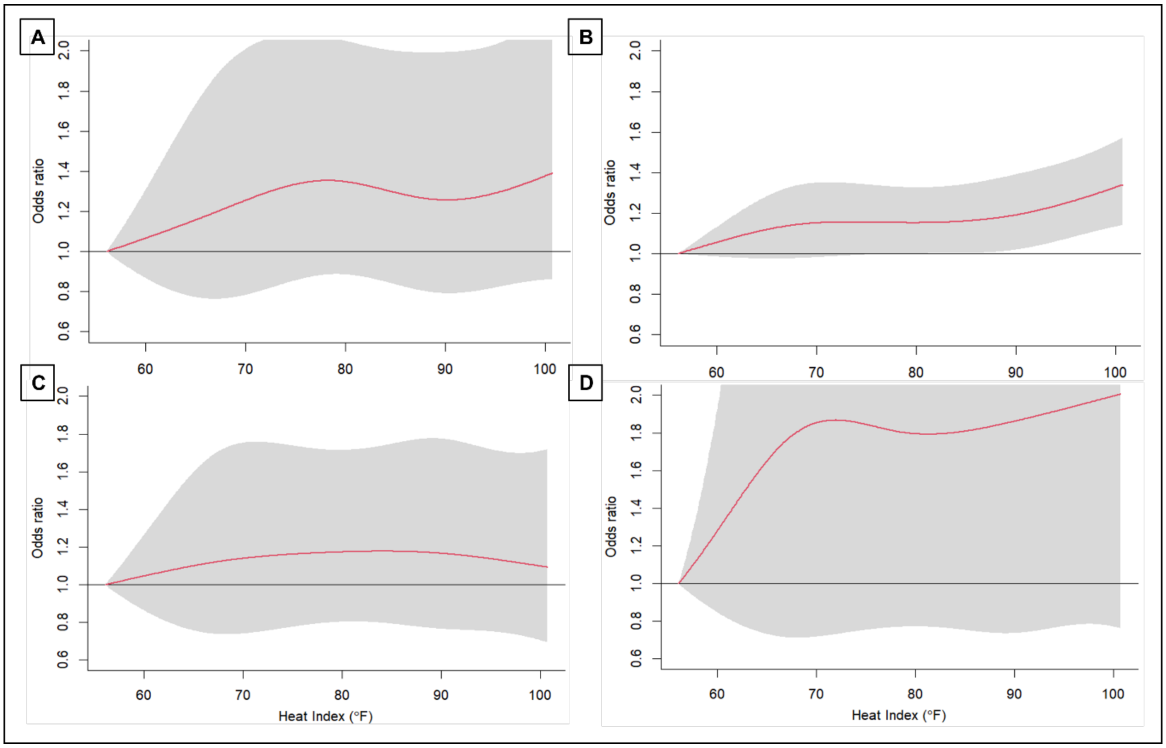

© 2026 Siau E et al. *JAMA Network Open*.

eFigure 15. Graph of cumulative odds (red curve) and 95% confidence intervals (gray margin) of all-cause ED-1 visits from May to September 2022-2024, among adults 65 years or older as a function of daily maximum heat index (HI_{max}) anomalies compared the reference value 0, representing the absence of anomalous HI_{max}. Odds ratio (OR) is displayed on the y-axis, and HI_{max} anomalies are displayed on the x-axis. Panels show models stratified by insurance type: private insurance (Panel A), Medicare (Panel B), Medicaid (Panel C), and missing insurance (Panel D).

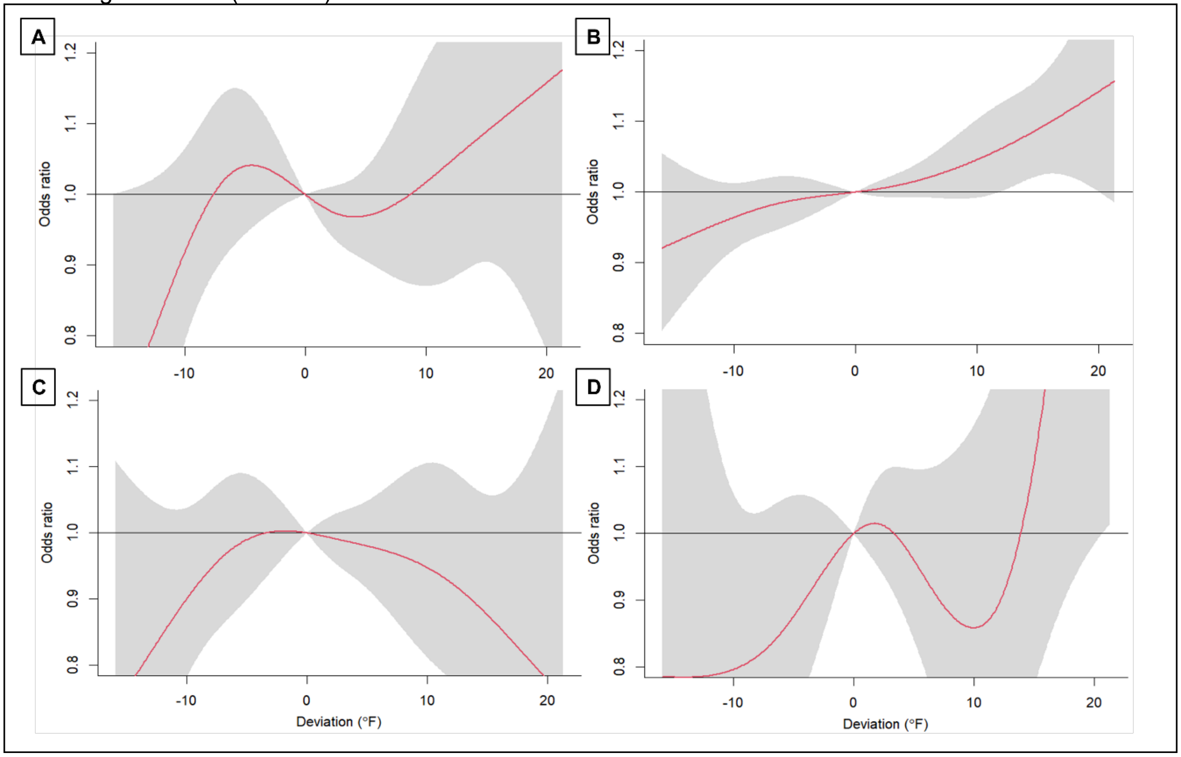

© 2026 Siau E et al. *JAMA Network Open*.

eFigure 16. Graph of cumulative odds (red curve) and 95% confidence intervals (gray margin) of all-cause ED-2 visits from May to September 2022-2024, among adults 65 years or older as a function of daily maximum heat index (HI_{max}) compared to the reference lowest HI_{max} of minimum effect (56.1°F). Odds ratio (OR) is displayed on the y-axis, and HI_{max} is displayed on the x-axis. Panels show models stratified by insurance type: private insurance (Panel A), Medicare (Panel B), Medicaid (Panel C), and missing insurance (Panel D).

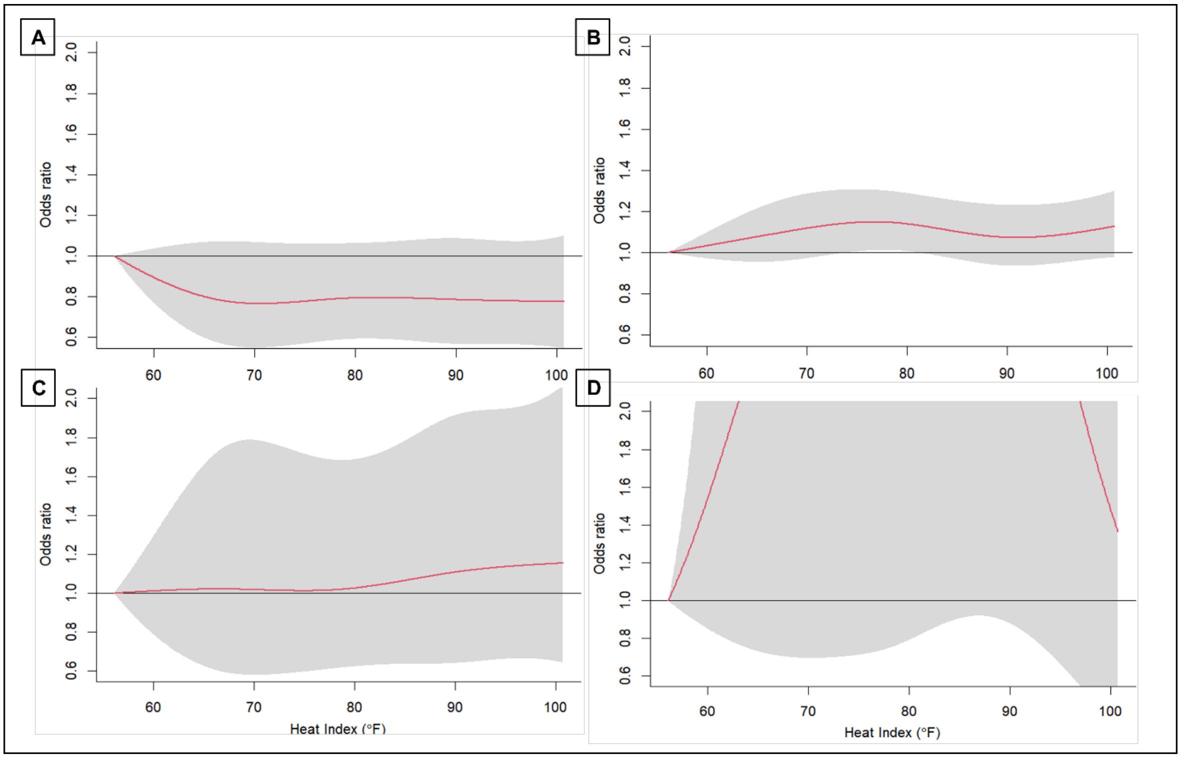

© 2026 Siau E et al. *JAMA Network Open*.

eFigure 17. Graph of cumulative odds (red curve) and 95% confidence intervals (gray margin) of all-cause ED-2 visits from May to September 2022-2024, among adults 65 years or older as a function of daily maximum heat index (HI_{max}) anomalies compared the reference value 0, representing the absence of anomalous HI_{max}. Odds ratio (OR) is displayed on the y-axis, and HI_{max} anomalies are displayed on the x-axis. Panels show models stratified by insurance type: private insurance (Panel A), Medicare (Panel B), Medicaid (Panel C), and missing insurance (Panel D).

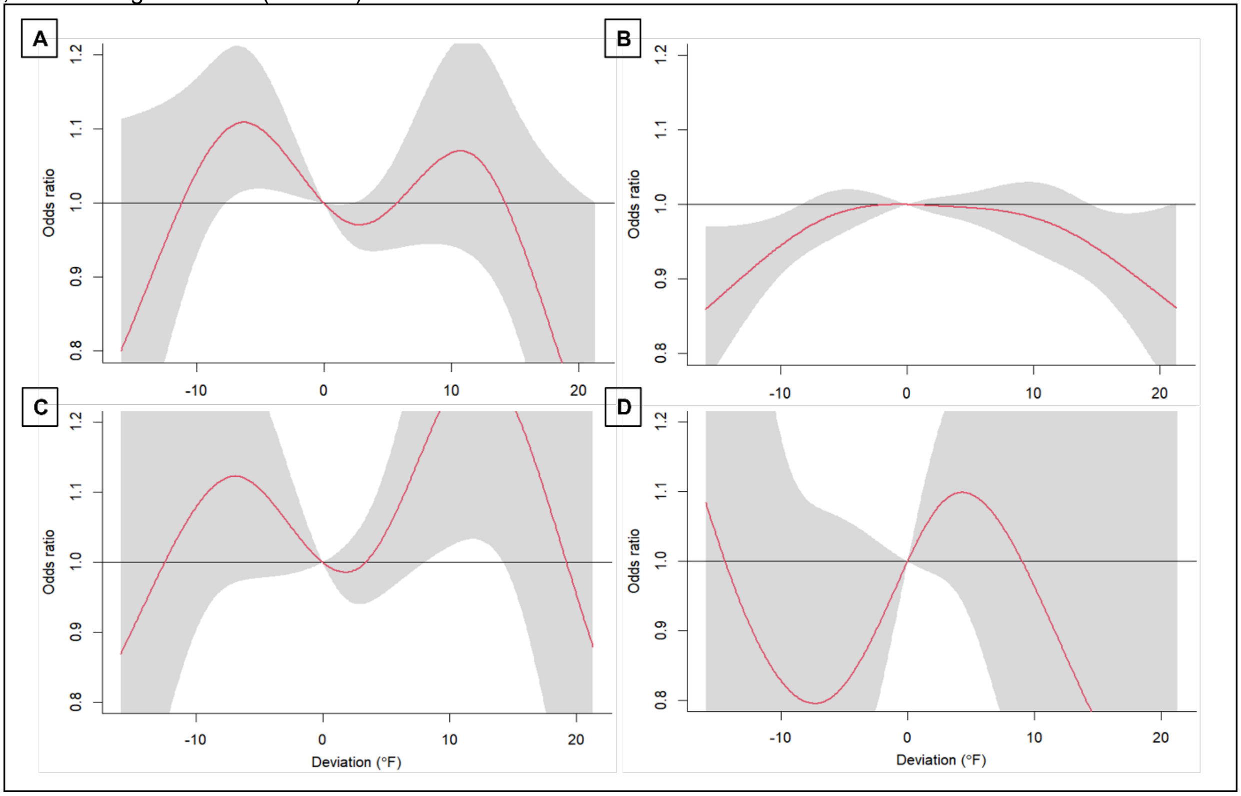

© 2026 Siau E et al. *JAMA Network Open*.

eFigure 18. Graph of cumulative odds (red curve) and 95% confidence intervals (gray margin) of all-cause ED visits from May to September 2022-2024, among adults 65 years or older as a function of daily maximum heat index (HI_{max}) compared to the reference lowest HI_{max} of minimum effect (56.1°F). Odds ratio (OR) is displayed on the y-axis, and HI_{max} is displayed on the x-axis. Panels A-D show ED-1 models stratified by race and ethnicity: non-Hispanic White (Panel A), non-Hispanic Black (Panel B), Hispanic or Latino (Panel C), and non-Hispanic Asian (Panel D). Panels E-H show ED-2 models stratified by race and ethnicity: non-Hispanic White (Panel E), non-Hispanic Black (Panel F), Hispanic or Latino (Panel G), and non-Hispanic Asian (Panel H). Models were not stratified by Non-Hispanic Native Hawaiian or Other Pacific Islander, Non-Hispanic American Indian or Alaska Native, or non-Hispanic Not Specified due to limited sample size.

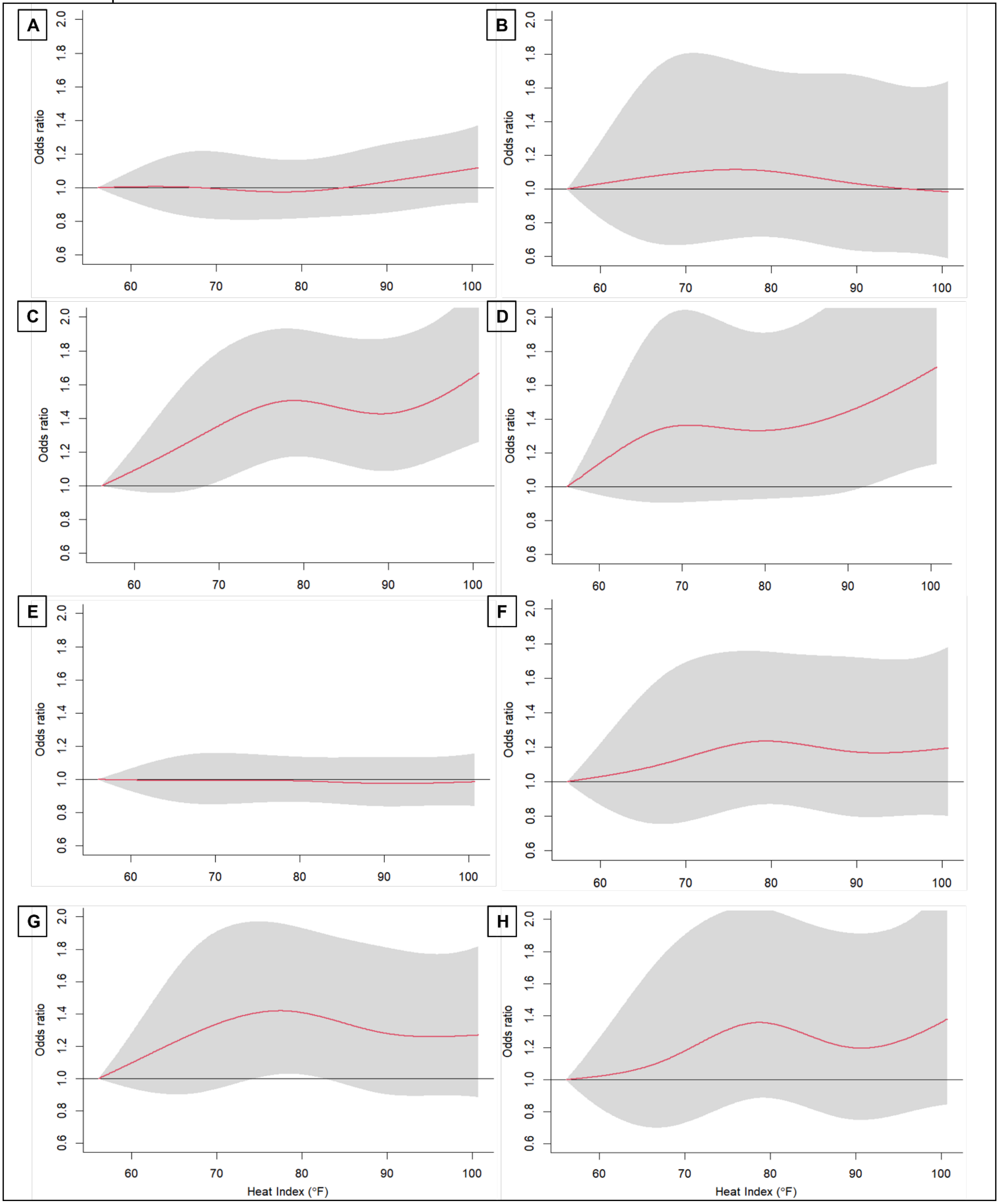

© 2026 Siau E et al. *JAMA Network Open*.

eFigure 19. Graph of cumulative odds (red curve) and 95% confidence intervals (gray margin) of all-cause ED visits from May to September 2022-2024, among adults 65 years or older as a function of daily maximum heat index (HI_{max}) anomalies compared the reference value 0, representing the absence of anomalous HI_{max}. Odds ratio (OR) is displayed on the y-axis, and HI_{max} anomalies are displayed on the x-axis. Panels A-D show ED-1 models stratified by race and ethnicity: non-Hispanic White (Panel A), non-Hispanic Black (Panel B), Hispanic or Latino (Panel C), and non-Hispanic Asian (Panel D). Panels E-H show ED-2 models stratified by race and ethnicity: non-Hispanic White (Panel E), non-Hispanic Black (Panel F), Hispanic or Latino (Panel G), and non-Hispanic Asian (Panel H). Models were not stratified by Non-Hispanic Native Hawaiian or Other Pacific Islander, Non-Hispanic American Indian or Alaska Native, or non-Hispanic Not Specified due to limited sample size.

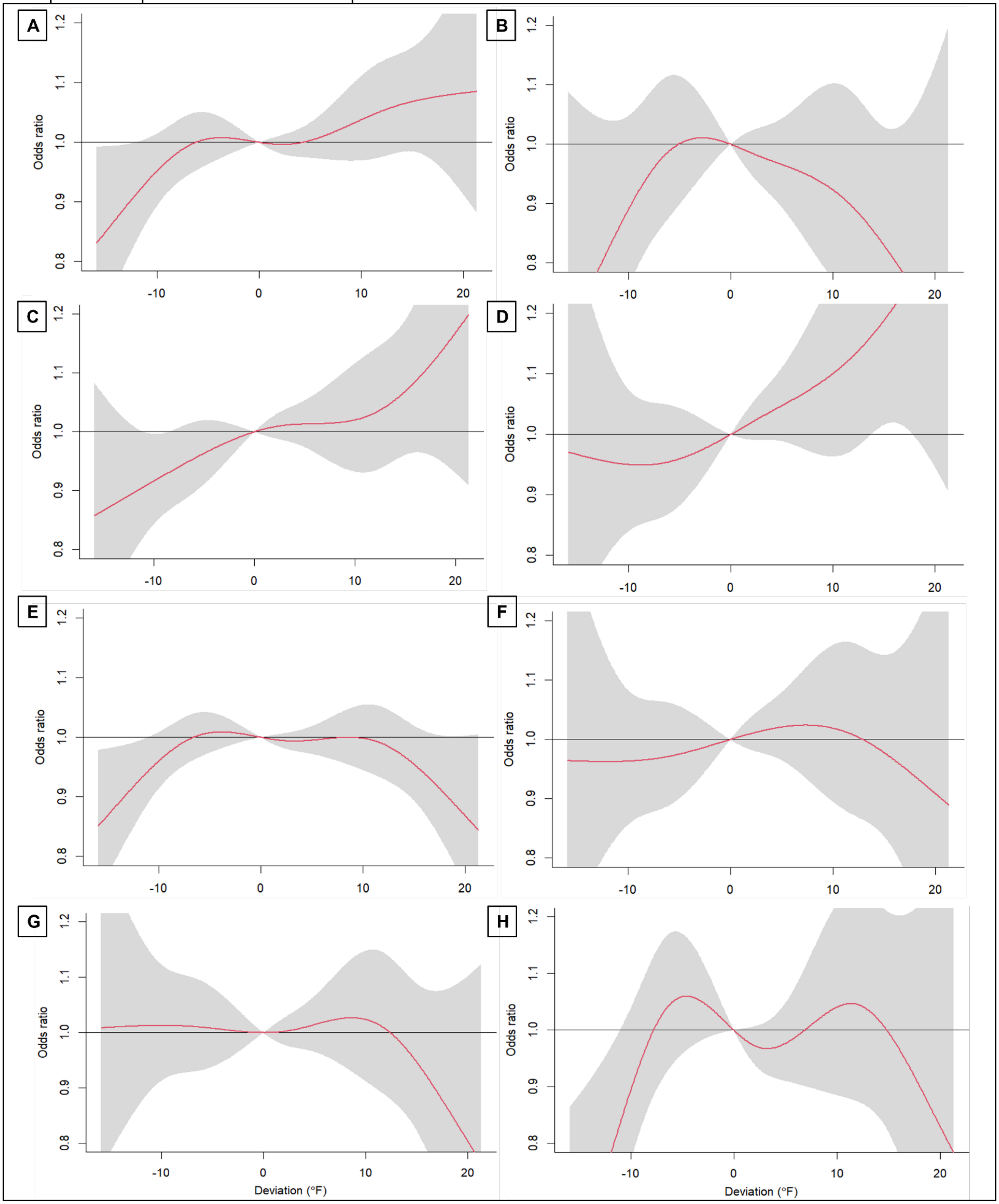

© 2026 Siau E et al. *JAMA Network Open*.

© 2026 Siau E et al. *JAMA Network Open*.

eFigure 20. Graph of cumulative odds (red curve) and 95% confidence intervals (gray margin) of all-cause ED visits from May to September 2022-2024, among adults 65 years or older as a function of daily maximum air temperature. Odds ratio (OR) is displayed on the y-axis, and daily maximum air temperature is displayed on the x-axis. Panels A and B show ED-1-based models stratified by sex: male (Panel A) and female (Panel B). Panels C and D show ED2-based models stratified by sex: male (Panel C) and female (Panel D).

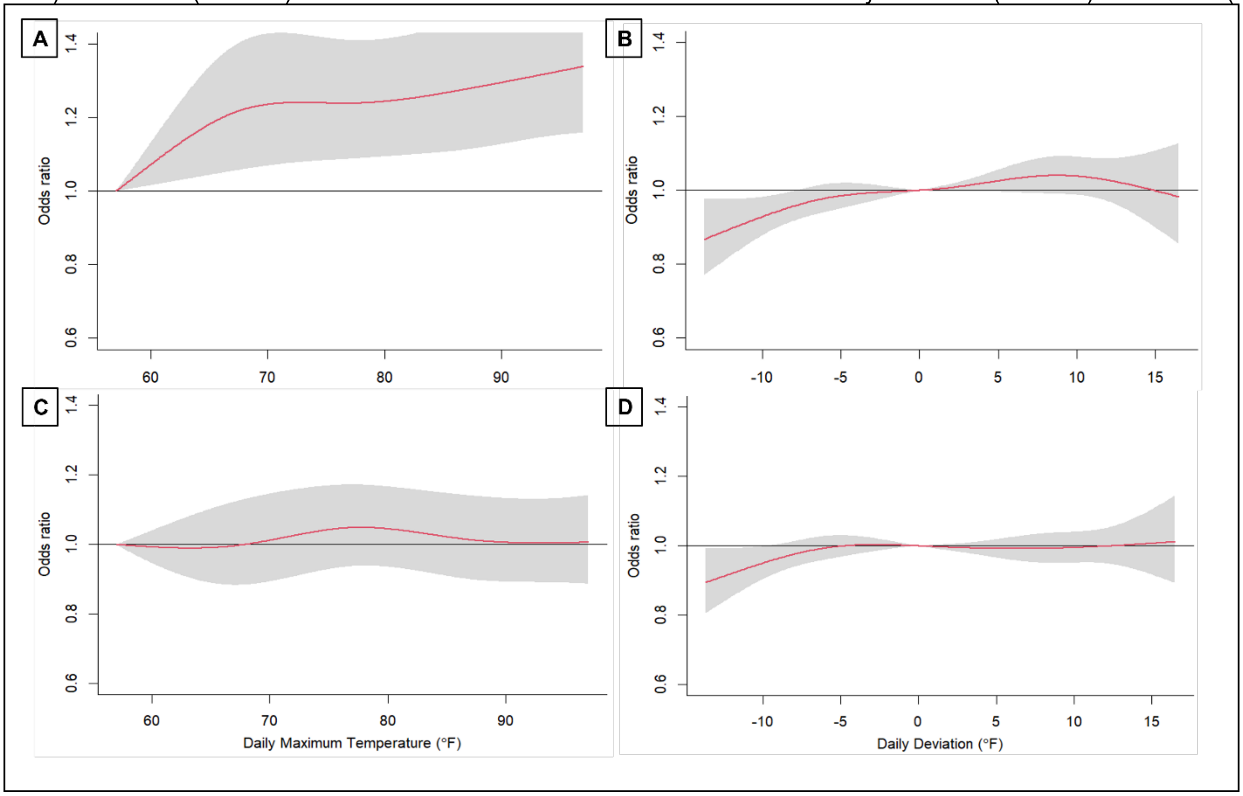

Supplement: Supplement 1. — eTable 1. Odds of daily maximum heat index (HImax) associated all-cause ED visits overall, at ED-1, and ED-2 eTable 2. Odds of daily HImax anomaly associated all-cause ED visits overall, at ED-1, and ED-2 eTable 3. Number of days and ED-1 visits across high HImax and HImax anomaly thresholds eFigure 1. Autocorrelative function analysis eFigure 2. Spearman correlation analysis of daily air quality index and all-cause ED visits eFigure 3. ED-1 models with HImax modeled with included quadratic term and varied spline flexibility eFigure 4. ED-1 models with HImax anomalies modeled with included quadratic term and varied spline flexibility eFigure 5. ED-2 models with HImax modeled with included quadratic term and varied spline flexibility eFigure 6. ED-2 models with HImax anomalies modeled with included quadratic term and varied spline flexibility eFigure 7. HImax models with alternative internal knot placements eFigure 8. HImax anomaly models with alternative internal knot placements eFigure 9. Models excluding individuals seen at both ED sites eFigure 10. HImax models stratified by patient sex eFigure 11. HImax anomaly models stratified by patient sex eFigure 12. HImax models stratified by patient age eFigure 13. HImax anomaly models stratified by patient age eFigure 14. ED-1 models as a function of HImax stratified by patient insurance eFigure 15. ED-1 models as a function of HImax anomalies stratified by patient insurance eFigure 16. ED-2 models as a function of HImax stratified by patient insurance eFigure 17. ED-2 models as a function of HImax anomalies stratified by patient insurance eFigure 18. HImax models stratified by patient self-reported race/ ethnicity eFigure 19. HImax anomaly models stratified by patient self-reported race/ ethnicity eFigure 20. Models with substituted daily maximum ambient temperature (Tmax) as the exposure [file jamanetwopen-e262645-s001.pdf]
