# Supplementary material for: Extreme Urban Heat and Emergency Department Visits in Older Adults
Source: JAMA Netw Open. 2026 Mar 20;9(3):e262645. doi: 10.1001/jamanetworkopen.2026.2645 (PMC13005158; doi:10.1001/jamanetworkopen.2026.2645)
Supplement: Supplement 2. — Data Sharing Statement [file jamanetwopen-e262645-s002.pdf]

Data Sharing Statement

Data
Data available: Yes
Data types: Data (not involving human participants), Data dictionary
How to access data: alexander.azan@nyulangone.org
When available: With publication

Supporting Documents
Document types: Statistical/analytic code
How to access documents: alexander.azan@nyulangone.org
When available: With publication

Additional Information
Who can access the data: researchers whose proposed use of the data has been approved
Types of analyses: for any purpose approved by the study team
Mechanisms of data availability: with a signed data access agreement
